# Expanding the Mutation Spectrum of Non-Syndromic Retinitis Pigmentosa in Consanguineous Pakistani Families: Unraveling Novel Pathogenic Variants in *RP1, PDE6B*, and *PRCD* Genes for Precision Diagnosis

**DOI:** 10.3390/genes17050529

**Published:** 2026-04-29

**Authors:** Tayyaba Shan, Nimra Mukhtar, Sayyed Hammad Ullah, Asad Ullah, Asfandyar Ahmad Khan, Yumei Li, Meng Wang, Raeesa Tehreem, Amtul Aziz, Kiran Afshan, Rui Chen, Sabika Firasat

**Affiliations:** 1 Department of Zoology, Faculty of Biological Sciences, Quaid-i-Azam University, University Road, Islamabad 45320, Pakistan; 2 Department of Ophthalmology, Center for Translational Vision Research, Irvine School of Medicine, University of California, Irvine, CA 92697, USA; 3 Department of Biological Sciences, Rawalpindi Medical University, Rawalpindi 46000, Pakistan; 4 Diagnostic Center, Al-Shifa Trust Eye Hospital, Jhelum Road, Rawalpindi 46000, Pakistan; 5 Department of Molecular and Human Genetics, Baylor College of Medicine, Houston, TX 77030, USA

**Keywords:** retinitis pigmentosa, non-syndromic, retinal dystrophies, novel, next-generation sequencing

## Abstract

**Background**: Non-syndromic retinitis pigmentosa (RP) is characterized by rod–cone degeneration, resulting in night blindness, visual field constriction, and eventual blindness. Recessively inherited RP is predominantly exacerbated in consanguineous populations, such as Pakistan. This study aimed to perform the genetic analysis of sixteen non-syndromic RP segregating Pakistani families, and to summarize the mutation spectrum of non-syndromic RP in our population by reviewing related literature. **Methods**: We screened 16 non-syndromic RP families using targeted capture panel sequencing of 344 genes related to inherited retinal dystrophies. Variants were prioritized based on rarity (minor allele frequency (MAF) < 0.001 in the gnomAD South Asian subset), pathogenicity assessments using ACMG/AMP criteria, and REVEL scores (>0.5). Candidate variants were validated for familial segregation through Sanger sequencing. **Results**: We identified 15 distinct variants across 14 genes associated with non-syndromic retinitis pigmentosa, comprising 6 missense, 7 nonsense, 1 frameshift, and 2 splice-site variants, including 4 novel variants, i.e., p.(Val220Met) and p.(Pro1282SerfsTer2) in *RP1*, 1 each in *PDE6B* (c.2021+5G>A), and *PRCD* p.(Ser38Ter). Homozygosity predominated, underscoring the impact of consanguinity on the burden of autosomal recessive disease in the present cohort, while the *CERKL* disease-causing mutation, i.e., p.(Arg257Ter), recurred in two families. **Conclusions**: This study expands Pakistan’s non-syndromic RP mutational spectrum by identifying novel variants in *RP1, PDE6B*, and *PRCD,* alongside recurrent *CERKL* and *RHO* mutations of the local population. The literature review suggests that *RP1, TULP1,* and *PDE6B* are among the most mutated genes in our population, supporting the value of population-specific genetic panels to enhance diagnostics and carrier screening.

## 1. Introduction

Non-syndromic retinitis pigmentosa (RP) is a leading cause of inherited retinal degeneration, characterized by progressive photoreceptor dysfunction primarily affecting rods and eventually cones. The global prevalence of RP is approximately 1 in 3000–4000, and over 3000 mutations have been reported in more than 80 genes, reflecting marked genetic heterogeneity of RP [1,2,3]. Clinically, RP is diagnosed via fundoscopy showing bony spicules pigmentation and arteriolar attenuation, visual field constriction, and where available, electroretinography (ERG) with reduced rod/cone responses, and optical coherence tomography (OCT) for retinal thinning [4,5]. Non-syndromic RP can follow autosomal recessive (ARRP), autosomal dominant (ADRP), X-linked (XLRP), or mitochondrial inheritance patterns, with disruptions in essential pathways, such as phototransduction, ciliary transport, retinal homeostasis, and structural integrity [5,6]. The advent of next-generation sequencing technologies has revolutionized the identification of causative variants across these inheritance modes, enabling the discovery of known and novel pathogenic mutations and disease-associated genes [7]. Nevertheless, diagnostic yields remain incomplete, with about 25% of ARRP cases unresolved in diverse cohorts, highlighting the need for continued investigation and improved population-specific variant data to inform gene therapy and precision medicine [8,9,10].

In consanguineous populations, such as those in Pakistan, where endogamous marriages are culturally prevalent (rates exceeding 60%), the incidence of ARRP is increased due to increased autozygosity [11]. Epidemiological studies estimate RP prevalence in Pakistan at 1 in 1000–2000, markedly higher than global averages, with diverse inheritance patterns observed, although these estimates are based on limited regional data [11,12]. Previous studies using Pakistani families have identified population-specific variants across different inheritance patterns. These findings have expanded the mutation spectrum and highlighted ethnic differences compared to European and East Asian populations [13,14,15]. However, a substantial proportion of inherited RP cases in Pakistan remain genetically unresolved, owing to infrastructural limitations, limited clinical characterization, restricted access to advanced genomic technologies in public laboratories, and research funding priorities skewed toward infectious diseases, leaving significant gaps in the understanding of the national mutational spectrum [16,17]. Large-scale studies are, therefore, essential to uncover rare variants across all inheritance patterns and support carrier screening programs in high-risk communities [18,19,20]. Non-syndromic retinitis pigmentosa represents the most genetically heterogeneous and prevalent IRD subtype in consanguineous populations [21,22]. In such genetically heterogeneous conditions, well-curated targeted panels provide a cost-effective and analytically reliable alternative to whole-exome sequencing, particularly in large consanguineous cohorts, where the majority of pathogenic variants reside in known disease genes [23]. Therefore, the present study employed a comprehensive, up-to-date retinal dystrophy gene panel while maximizing variant detection accuracy and interpretability [15].

Building on this foundation, the present study pursues two key objectives: first, to leverage NGS to perform panel sequencing of Pakistani families affected with non-syndromic RP and, second, to conduct a targeted narrative literature review of previously reported mutations in non-syndromic RP genes from Pakistani populations to summarize frequently mutated genes and prevalent mutations. By integrating novel and known variants into the existing genetic framework, we aim to broaden the currently reported variant spectrum and provide population-relevant data as a requisite to precision medicine.

## 2. Materials and Methods

### 2.1. Patient Enrollment and Sample Preparation

This study received ethical approval from the Bio-Ethical Review Committee of the Faculty of Biological Sciences at Quaid-i-Azam University, Islamabad, Pakistan (protocol # BEC-FBS-QAU2023-491), and the Ethical Review Committee, Al-Shifa Trust, Rawalpindi, Pakistan (Reference No. ERC-09/AST-23), adhering strictly to the Declaration of Helsinki guidelines. A total of 16 consanguineous Pakistani families segregating non-syndromic RP were recruited following comprehensive ophthalmic evaluations by specialized clinicians at Al-Shifa Trust Eye Hospital. Diagnostic criteria included detailed pedigree analysis, medical history review, fundus photography where available, and visual acuity testing to confirm progressive photoreceptor degeneration. Although advanced investigations such as ERG were not available, the clinical presentation was consistent with established IRD phenotypes. Informed consent was obtained from all participants or guardians, emphasizing voluntary participation and data confidentiality. Venous blood samples (3–5 mL) were collected into EDTA-coated tubes from index cases and consenting relatives. Genomic DNA was isolated using an optimized organic extraction protocol involving proteinase K digestion and phenol-chloroform purification [15], followed by quality assessment through agarose gel electrophoresis at the Department of Zoology, Quaid-i-Azam University, Islamabad, Pakistan.

### 2.2. Genomic Sequencing

Targeted panel sequencing was performed on genomic DNA obtained from two affected individuals per family at the Advanced Genomics Facility, Baylor College of Medicine, Houston, TX, USA. Library preparation was carried out using the KAPA HyperPlus Kit (Roche, Basel, Switzerland) according to the manufacturer’s protocol. Libraries were multiplexed and enriched using a hybridization-based capture approach targeting a curated panel of 344 genes known to be associated with inherited retinal dystrophies, employing the SureSelect XT HS system (Agilent Technologies, Santa Clara, CA, USA), optimized for Illumina sequencing platforms. The gene panel was custom-designed based on a comprehensive compilation of genes previously implicated in inherited retinal dystrophies, curated from publicly available databases and literature, including RetNet, OMIM, and peer-reviewed studies. The selection criteria prioritized genes with established or strongly suspected roles in retinal degeneration. The panel content was finalized prior to sequencing and remained consistent throughout the study period (Supplementary Table S1), as described previously by Tehreem et al. [15]. Post-capture libraries underwent standard quality control assessments, including fragment size distribution and concentration measurements, and were sequenced on a NovaSeq 6000 platform (Illumina).

**Figure 1:**
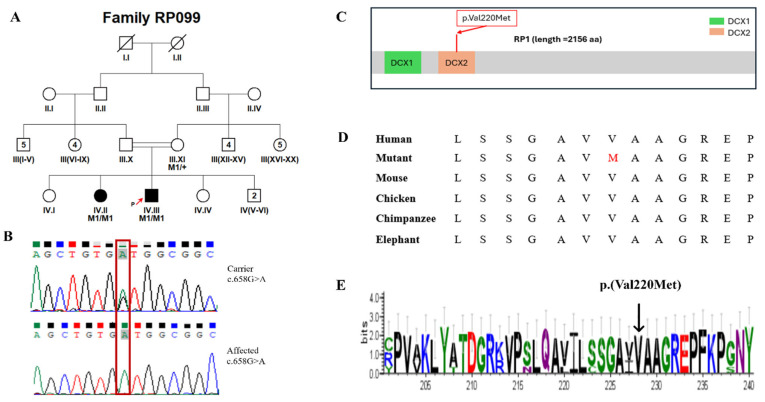
(**A**) Pedigree of the RP099 family. Affected individuals are indicated by filled symbols, while unaffected individuals are shown as open symbols. Squares represent males and circles represent females. Slashes denote deceased individuals. Double horizontal lines indicate consanguineous unions. Generations are labeled with Roman numerals, and individuals within each generation are numbered. Numbers in parentheses indicate the number of individuals. The proband is indicated by a red arrow. (**B**). Sanger sequencing chromatograms showing the identified variant in carrier and affected individuals. The variant position is highlighted by a red box. (**C**) Linear domain map of the RP1 protein (2156 aa) depicting novel missense variant p.(Val220Met) (located in the DCX2 domain) identified in this study. (**D**) ClustalW multiple sequence alignment of RP1 across species (human, mutant, mouse, chicken, chimpanzee, and elephant), focusing on the conserved motif (LSGAVMAAGREP) and the p.(Val220Met) substitution. (**E**) Weblogo showing sequence conservation across closely related species.

**Figure 2:**
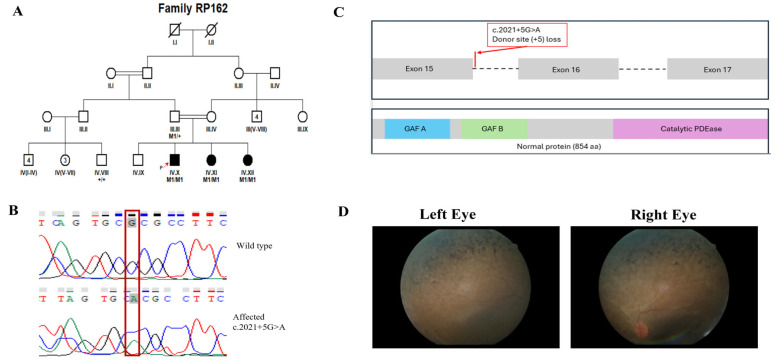
(**A**) Pedigree of the RP162 family, affected individuals are indicated by filled symbols, while unaffected individuals are shown as open symbols. Squares represent males and circles represent females. Slashes denote deceased individuals. Double horizontal lines indicate consanguineous unions. Generations are labeled with Roman numerals, and individuals within each generation are numbered. Numbers in parentheses indicate the number of individuals. The proband is indicated by a red arrow. (**B**) Sanger sequencing chromatograms confirming the detected variant. The variant position is highlighted by a red box. (**C**) Linear domain map of the PDE6B protein illustrating the novel variant: the splice site mutation c.2021+5G>A. (**D**) Fundus photograph of the family’s proband.

**Figure 3:**
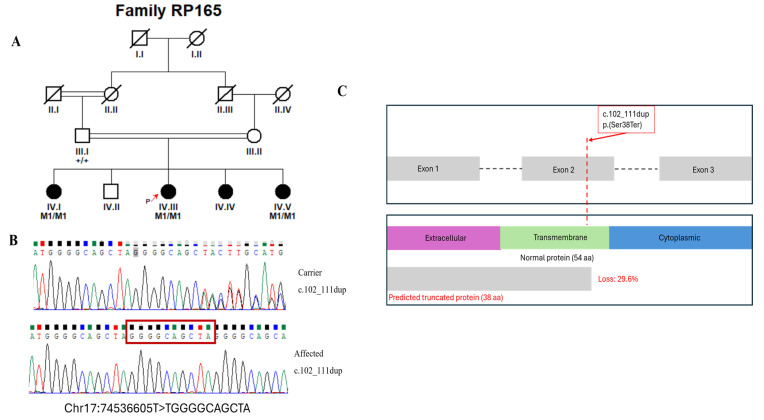
(**A**) Pedigree of the RP165 family, affected individuals are indicated by filled symbols, while unaffected individuals are shown as open symbols. Squares represent males and circles represent females. Slashes denote deceased individuals. Double horizontal lines indicate consanguineous unions. Generations are labeled with Roman numerals, and individuals within each generation are numbered. Numbers in parentheses indicate the number of individuals. The proband is indicated by a red arrow. (**B**) Sanger sequencing chromatograms confirming the detected variant. The variant position is highlighted by a red box. (**C**) Linear domain map of the PRCD protein illustrating the novel variant: the nonsense mutation p.(Ser38Ter) (potentially resulting in a truncated protein and loss of function).

**Figure 4:**
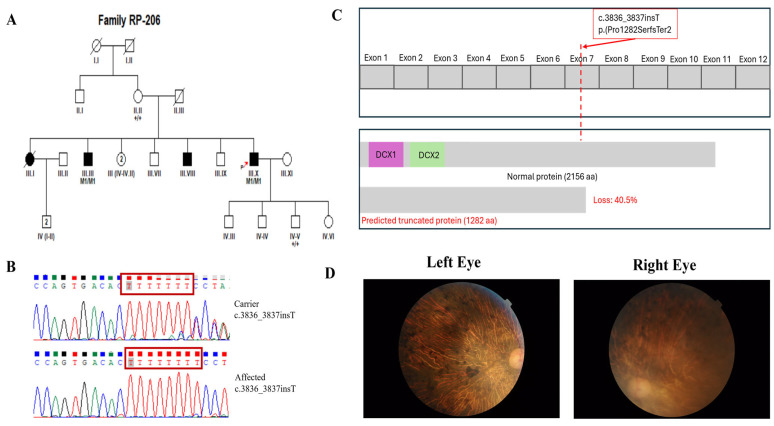
(**A**) Pedigree of the RP206 family, affected individuals are indicated by filled symbols, while unaffected individuals are shown as open symbols. Squares represent males and circles represent females. Slashes denote deceased individuals. Double horizontal lines indicate consanguineous unions. Generations are labeled with Roman numerals, and individuals within each generation are numbered. Numbers in parentheses indicate the number of individuals. The proband is indicated by a red arrow. (**B**) Sanger sequencing chromatograms confirming the detected variant. The variant position is highlighted by a red box. (**C**) Linear domain map of the RP1 protein illustrating the novel variant: the frameshift variant p.(Pro1282SerfsTer2). (**D**) Fundus photograph of proband of family RP206.

Sequencing was performed to achieve a mean on-target coverage exceeding 100× across the panel. The capture design included all protein-coding exons and flanking intronic splice junctions (±20 bp) of the 344 IRD-associated genes, with >99% of targeted regions covered at a minimum depth of 20×. Regions exhibiting suboptimal coverage were manually inspected, and clinically relevant low-coverage exons were subsequently analyzed using targeted Sanger sequencing. For each family, two affected individuals were selected for targeted panel sequencing where available, to increase confidence in variant identification and reduce the likelihood of false-positive findings. Selection was based on clear clinical diagnosis and availability of high-quality DNA samples. Following identification of candidate variants, segregation analysis was performed by Sanger sequencing in additional available family members, including both affected and unaffected individuals. The number of genotyped individuals per pedigree varied depending on family size and sample availability. In all families, the identified variants were assessed for co-segregation with the disease phenotype across the genotyped individuals. All reported candidate variants showed segregation patterns consistent with the expected mode of inheritance within each pedigree. Genotype status was recorded as homozygous mutant (M1/M1), heterozygous (M1/+), or wild type (+/+), and is indicated in the corresponding pedigrees (Figure 1, Figure 2, Figure 3 and Figure 4 and Supplementary Figure S1).

### 2.3. Bioinformatic Processing and Variant Analysis

Raw sequencing reads were aligned to the human reference genome (GRCh37/hg19) using BWA-MEM. Duplicate reads were marked, and base quality score recalibration was performed following GATK best-practice guidelines. Variant calling was conducted using GATK HaplotypeCaller, and identified variants were annotated using ANNOVAR (version 2018Apr16). Quality control filters were applied to retain high-confidence variants, including a minimum read depth of ≥20×, genotype quality ≥ 20, and appropriate allele balance consistent with the expected mode of inheritance. Although the mean sequencing depth exceeded 100×, all reported pathogenic, likely pathogenic, and variants of uncertain significance (VUS) were supported by ≥30× coverage at the variant position.

Variant prioritization focused on rare variants with a minor allele frequency ≤ 0.01 in population databases, including gnomAD, ExAC, and the 1000 Genomes Project, with more stringent thresholds (≤0.001) applied for autosomal recessive models. Only protein-altering variants, including nonsense, frameshift, canonical splice-site, and missense variants, were retained for downstream analysis. Missense variants were further evaluated using multiple in silico prediction tools, including SIFT, PolyPhen-2, REVEL, CADD, MutationTaster, and SpliceAI, along with assessments of evolutionary conservation. Variant classification was performed using the ACMG/AMP 2015 framework, with interpretation guided by subsequent updates and refinements, including ClinGen Sequence Variant Interpretation (SVI) Working Group recommendations for loss-of-function, splice-site, and missense variants. Previously reported variants were cross-referenced against HGMD, ClinVar, and LOVD databases, while novel variants were assessed based on rarity, predicted functional impact, segregation within families, and consistency with the clinical phenotype. All candidate variants classified as pathogenic, likely pathogenic, or VUS were validated by Sanger sequencing using an ABI 3500 Genetic Analyzer (Applied Biosystems, Thermo Fisher Scientific, Waltham, MA, USA), with primers designed using Primer-BLAST (NCBI) to ensure specificity.

### 2.4. Review of Literature

A targeted narrative literature review was conducted to contextualize the findings of the present study and to summarize previously reported genetic variants associated with non-syndromic retinitis pigmentosa in the Pakistani population. PubMed and Google Scholar were searched using a targeted strategy and combinations of the keywords “non-syndromic retinitis pigmentosa,” “retinitis pigmentosa,” “Pakistan,” and individual gene names identified in this study. Articles published in English up to July 2025 were considered.

Inclusion criteria comprised original research articles reporting molecular characterization of Pakistani RP cases, including mutation reports, cohort-based genetic studies, and family-based case series with clearly described clinical phenotypes and genetic findings. Studies were excluded if they (i) lacked molecular confirmation of variants, (ii) involved non-Pakistani populations, or (iii) were review articles without primary genetic data. Case reports were included only when they provided novel or well-documented pathogenic variants with sufficient clinical detail. To minimize redundancy, duplicate reports describing the same families or variants were identified through comparison of pedigree information, geographic origin, and variant details, and were counted only once. The extracted data included gene name, variant type, inheritance pattern, and clinical phenotype. Given the narrative scope of the review, no PRISMA workflow or quantitative study selection tracking was applied. While clinical assessment and genetic screening approaches varied across studies, most of these studies relied on standardized ophthalmic evaluations (i.e., fundoscopy), linkage analysis followed by Sanger sequencing, and next-generation sequencing-based methods. This approach helped us gather population-specific genetic evidence while recognizing differences in study design, offering a general overview rather than a formal systematic review.

## 3. Results

### 3.1. Clinical Phenotype

In this study, we recruited 16 consanguineous Pakistani families segregating non-syndromic RP through expert ophthalmologists at Al-Shifa Trust Eye Hospital, Rawalpindi, Pakistan. The RP cohort reflected Pakistan’s ethnic diversity, comprising ten families of Punjabi origin (RP005, RP010, RP024, RP049, RP060, RP099, RP170, RP172, RP174, and RP206), three Pakhtun families (RP162, RP165, and RP244), two Muhajir families (RP076 and RP168), and one Saraiki family (RP189). Detailed clinical features at enrollment are summarized in Table 1, and pedigrees are shown in Figure 1, Figure 2, Figure 3 and Figure 4 and Supplementary Figure S1. Family interviews recorded ethnicity, age at symptom onset, and disease progression.

Age at onset varied, ranging from early-onset or congenital presentation in seven families (RP049, RP076, RP170, RP172, RP174, RP189, and RP244) to childhood or adolescent onset in others (9 years in RP162, 10 years in RP206, 12 years in RP024, 14 years in RP099, 15 years in RP005 and RP060, 20 years in RP165, and up to 25 years in RP168). Although “typical” RP often presents in adolescence, early-onset disease is well recognized in autosomal recessive IRDs and falls within the LCA/EOSRD-RP continuum. Across the cohort, night blindness was present in all affected individuals and represented the most consistent initial symptom.

Beyond night blindness, probands of some families exhibited cone-related symptoms at the time of enrollment, including hemeralopia (RP005, RP024, RP060, RP076, RP099, RP162, RP165, RP168, RP170, RP206, and RP244), photophobia (RP005, RP076, RP099, RP168, and RP244), and color vision deficits (RP024, RP076, RP099, RP162, RP165, RP168, RP170, RP206, and RP244). Refractive errors were also noted, including myopia (RP005 and RP099) and hyperopia (RP049 and RP076). No systemic abnormalities were identified in these families, supporting an isolated retinal phenotype.

### 3.2. Genetic Analyses

Genetic analyses identified 15 distinct variants across 14 genes associated with non-syndromic RP (Table 2). Among the 16 families, disease-causing variants in *CERKL* and *RP1* were identified in 2 families each, while variants in the remaining families were found in *SAG, RPE65, MERTK, IDH3B, PDE6B, PRCD, EYS, PDE6A, ACBD5, RHO, PROM1*, and *TTC8*, with 1 family for each gene (Table 2). The mutational spectrum across these genes comprised six missense variants, seven nonsense variants, two splice-site, and one frameshift variant. As expected, all the variants in our study were segregating in an autosomal recessive pattern. Four of the fifteen distinct alleles were not reported in gnomAD, while the rest were rare and showed no homozygous individuals in the database (Table 2 and Table 3). The four novel variants included p.(Val220Met) and p.(Pro1282SerfsTer2) in *RP1*, c.2021+5G>A; p.(?) in *PDE6B*, and p.(Ser38Ter) in *PRCD*. Interestingly, we identified one homozygous variant, c.769C>T, p.(Arg257Ter), of *CERKL* (NM_201548.5), segregating in two separate pedigrees (RP005 and RP172; Supplementary Figure S1).

**Figure 5:**
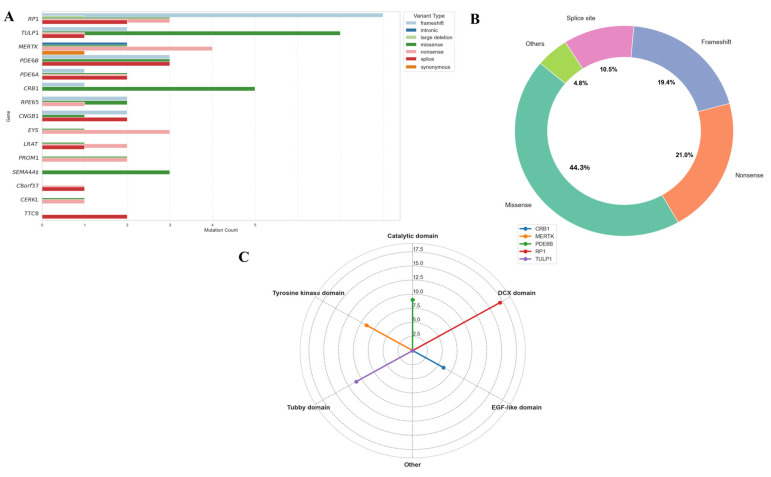
Genetic landscape of non-syndromic RP in Pakistani populations. (**A**) Illustrating the proportional frequency of genes, highlighting *RP1* and *TULP1* as the most frequently mutated genes. (**B**) Depicting the distribution of mutation types, with missense mutations predominant. (**C**) The radar plot illustrates the functional clustering of variants in the top five mutated genes in the Pakistani cohort, highlighting gene-specific mutation hotspots. ‡ Only RP-associated SEMA4A variants/studies were included.

Analysis of variant frequencies across genes, derived from a comprehensive compilation of previously reported mutations in non-syndromic RP within the Pakistani cohort (Table 4; Figure 5), revealed that *RP1* appears to be the most frequently mutated gene (14.0%), followed by *TULP1* (10.3%), *MERTK* (8.4%), *PDE6B* (7.5%), and *CRB1* and *PDE6A* (each 6.5%). Across the Pakistani cohort, missense variants predominated (42.1%), followed by frameshift (35.5%) and nonsense mutations (15.9%). A high prevalence of truncating variants (nonsense/frameshift, 51.4%) was observed in *PDE6B, RP1,* and *TULP1*. In the most frequently mutated genes of the Pakistani cohort, functional clustering was observed, with *RP1* mutations frequently affecting the DCX domain, *TULP1* variants clustering in the tubby domain, *MERTK* mutations disrupting the tyrosine kinase domain, *PDE6B* variants involving the catalytic domain, and *CRB1* missense changes concentrated in EGF-like domains (Figure 5C).

**Table 1:** Demographic and clinical features of probands of 16 consanguineous Pakistani RP families described in the current study.

Family ID	Proband ID	Age (in Years)		Ethnicity	No. of Affected Cases in Family	Nyctalopia	Hemeralopia	Photophobia	Color Perception Deficit	Myopia	Hyperopia	Others
		At Onset	At Enrollment									
RP005	IV-II	Juvenile	31	Punjabi	7	Yes	Yes	Yes	No	No	Yes	No
RP010	IV-II	N/A	N/A	Punjabi	5	Yes	Yes	N/A	N/A	N/A	N/A	No
RP024	IV-VI	Juvenile	29	Punjabi	3	Yes	Yes	No	Yes	No	Yes	No
RP049	IV-VII	Early onset	24	Punjabi	3	Yes	No	No	No	No	Yes	No
RP060	IV-I	Juvenile	23	Punjabi	4	Yes	Yes	No	Yes	No	No	No
RP076	III-I	Early onset	49	Muhajir	3	Yes	Yes	Yes	Yes	No	Yes	Cataract
RP099	IV-III	Juvenile	23	Punjabi	2	Yes	Yes	Yes	Yes	Yes	No	No
RP162	IV-X	Early onset	13	Pakhtun	3	Yes	Yes	No	Yes	No	N/A	No
RP165	IV-III	Late onset	30	Pakhtun	4	Yes	Yes	No	Yes	No	No	No
RP168	IV-XII	Late onset	57	Muhajir	2	Yes	Yes	Yes	Yes	No	No	No
RP170	IV-VII	Early onset	37	Punjabi	7	Yes	Yes	No	Yes	No	No	No
RP172	III-VII	Juvenile	47	Punjabi	5	Yes	Yes	No	Yes	No	N/A	No
RP174	IV-II	Early onset	4	Punjabi	3	Yes	Yes	No	Yes	No	No	No
RP189	IV-III	Early onset	12	Saraiki	3	Yes	Yes	No	Yes	No	No	No
RP206	III-VIII	Juvenile	57	Punjabi	6	Yes	Yes	Yes	No	No	No	No
RP244	IV-IX	Early onset	25	Pakhtun	6	Yes	Yes	Yes	Yes	No	No	No

Age at onset was categorized as early onset (<10 years), juvenile onset (10–20 years), and late onset (>20 years). Age at enrollment represents the age at the time of clinical evaluation. Clinical features were recorded based on patient history and ophthalmological examination. Nyctalopia refers to night blindness, and hemeralopia refers to day blindness. Color perception deficit indicates impaired color vision. Refractive errors are reported as myopia or hyperopia where available. N/A indicates data not available.

**Table 2:** List of genetic variants identified in 16 Pakistani families.

Family	Gene	Transcript ID	Site of Mutation	c.DNA Change	Protein Change	Variant Type	db SNP ID	ClinVar ID	ClinVar Report	ACMG Classification
RP005	*CERKL*	NM_201548.5	Chr2:182423344G>A	c.769C>T	p.(Arg257Ter)	Nonsense	rs121909398	2364	Pathogenic	consistent with ClinVar
RP010	*SAG*	NM_000541	Chr2:234243675C>T	c.874C>T	p.(Arg292Ter)	Nonsense	rs397514681	41897	Pathogenic	consistent with ClinVar
RP024	*TTC8*	NM_144596.4	Chr14:89300035A>G	c.115-2A>G	p.(?)	Splice site	rs587777809	2532	Likely pathogenic	consistent with ClinVar
RP049	*RPE65*	NM_000329	Chr1:68910541G>A	c.271C>T	p.(Arg91Trp)	Missense	rs61752871	12861	Pathogenic	consistent with ClinVar
RP060	*MERTK*	NM_006343.3	Chr2:112687071C>T	c.436C>T	p.(Gln146Ter)	Nonsense	rs1461788401	N/A	N/A	Likely Pathogenic [PVS1_very strong, PM2_moderate, PP1_supporting, PP4_supporting] [24]
RP076	*IDH3B*	NM_006899.5	Chr20:2640992A>G	c.685T>C	p.(Phe229Leu)	Missense	rs752597235	971237	VUS	consistent with ClinVar
RP099	*RP1*	NM_006269.2	Chr8:55534719G>A	c.658G>A	p.(Val220Met)	Missense	* Novel	N/A	N/A	VUS [PM1_supporting, PM2_moderate, PP1_supporting, PP3_supporting, PP4_supporting]
RP162	*PDE6B*	NM_000283.4	Chr4:657664G>A	c.2021+5G>A	p.(?)	Splice site	* Novel	N/A	N/A	VUS [PM2_supporting, PP3_supporting, PP1_supporting, PP4_supporting]
RP165	*PRCD*	NM_001077620.3	Chr17:74536605T> TGGGGCAGCTA	c.102_111dup	p.(Ser38Ter)	Nonsense	* Novel	N/A	N/A	Pathogenic [PVS1_very strong, PM2_supporting, PP3_supporting, PP4_supporting, PP1_supporting]
RP168	*EYS*	NM_001142800.2	Chr6:66204814G>A	c.490C>T	p.(Arg164Ter)	Nonsense	rs794727631	197186	Pathogenic	consistent with ClinVar
RP170	*PDE6A*	NM_000440.3	Chr5:149310680G>A	c.769C>T	p.(Arg257Ter)	Nonsense	rs146591309	437984	Pathogenic	consistent with ClinVar
RP172	*CERKL*	NM_201548.5	Chr2:182423344G>A	c.769C>T	p.(Arg257Ter)	Nonsense	rs121909398	2364	Pathogenic	consistent with ClinVar
RP174	*ACBD5*	NM_145698.5	Chr10:27512375C>T	c.382G>A	p.(Glu128Lys)	Missense	rs1217409899	N/A	N/A	VUS [PM2_supporting, PP3_supporting, PP1_supporting, PP4_supporting]
RP189	*RHO*	NM_000539.3	Chr3:129249805G>A	c.448G>A	p.(Glu150Lys)	Missense	rs104893791	13046	Pathogenic	consistent with ClinVar
RP206	*RP1*	NM_006269.2	Chr8:55540278C>CT	c.3836_3837insT	p.(Pro1282SerfsTer2)	Frameshift	* Novel	N/A	N/A	Likely pathogenic [PVS1_very strong, PM2_moderate, PP1_supporting, PP3_supporting, PP4_supporting]
RP244	*PROM1*	NM_006017.3	Chr4:15992882G>A	c.1919C>T	p.(Ser649Leu)	Missense	rs761911901	425331	VUS	consistent with ClinVar

* Indicates novel variants identified in this study and classified according to ACMG/AMP guidelines based on available evidence, including segregation data, in silico predictions, and population frequency. Variants without an asterisk have previously been reported and their clinical significance is based on ClinVar classification. ACMG/AMP criteria applied include PVS1 (pathogenic very strong; null variant in a gene where loss of function is a known disease mechanism), PM2 (pathogenic moderate; absent or extremely rare in population databases), PP1 (pathogenic supporting; co-segregation with disease in affected family members), PP3 (pathogenic supporting; multiple computational lines of evidence support a deleterious effect), and PP4 (pathogenic supporting; phenotype highly specific for the disease). ACMG/AMP classification with detailed evidence codes was applied only to novel variants identified in this study. For previously reported variants, clinical significance and ACMG classification are based on ClinVar submissions without independent reevaluation. “N/A” indicates that no corresponding entry available in ClinVar or other referenced databases for the given variant. Sequence variant nomenclature follows the Human Genome Variation Society (HGVS) recommendations and was verified using Mutalyzer (https://mutalyzer.nl/ (accessed on 15 January 2026)). Genomic coordinates are based on the GRCh37/hg19 reference assembly. Population frequency and prior variant reports were assessed using publicly available databases, including gnomAD, dbSNP, and ClinVar. Variants designated as “novel” are absent from these databases and have not been previously reported in the literature.

**Table 3:** In silico prediction and population frequency of identified variants in the present study.

Gene	Variant	Protein Change	gnomAD AF	SIFT	Polyphen2	REVEL	Mutation Taster	Mutation Accessor	Provean	DANN	CADD	SpliceAI
*CERKL*	NM_201548.5: c.769C>T	NP_963842.1: p.(Arg257Ter)	3.319 × 10^−4^	N/A	N/A	N/A	Disease causing	D	D	D	31	B
*SAG*	NM_000541: c.874C>T	NP_000532.2: p.(Arg292Ter)	4.816 × 10^−5^	N/A	N/A	N/A	Disease causing	N/A	N/A	D	38	B
*TTC8*	NM_144596.4: c.115-2A>G	NP_653197.2: p.(?)	N/A	N/A	N/A	N/A	Disease causing	N/A	N/A	D	32	B
*RPE65*	NM_000329: c.271C>T	NP_000320.1: p.(Arg91Trp)	5.308 × 10^−5^	D (0.012)	D	0.85	Disease causing	Med	D	D	27.9	M
*MERTK*	NM_006343.3: c.436C>T	NP_006334.2: p.(Gln146Ter)	3.98 × 10^−6^	N/A	N/A	N/A	Disease causing	N/A	D	D	37	B
*IDH3B*	NM_006899.5: c.685T>C	NP_008830.2: p.(Phe229Leu)	1.193 × 10^−4^	D	D	0.78	Disease causing	Hi	D	D	28.9	B
*RP1*	NM_006269.2: c.658G>A	NP_006260.1: p.(Val220Met)	N/A	D (0.001)	D	0.88	Disease causing	Med	D	D	27.7	B
*PDE6B*	NM_000283.4: c.2021+5G>A	NP_000274.3: p.(?)	N/A	N/A	N/A	N/A	Disease causing	D	N/A	N/A	18.12	D
*PRCD*	NM_001077620.3: c.102_111dup	NP_001071088.1: p.(Ser38Ter)	N/A	N/A	N/A	N/A	Disease causing	N/A	N/A	N/A	N/A	N/A
*EYS*	NM_001142800.2: c.490C>T	NP_001136272.1: p.(Arg164Ter)	7.961 × 10^−6^	N/A	N/A	N/A	Disease causing	D	D	D	34	B
*PDE6A*	NM_000440.3: c.769C>T	NP_000431.2: p.(Arg257Ter)	3.89 × 10^−5^	N/A	N/A	N/A	Disease causing	D	D	D	36	B
*CERKL*	NM_201548.5: c.769C>T	NP_963842.1: p.(Arg257Ter)	3.319 × 10^−4^	N/A	N/A	N/A	Disease causing	D	D	D	31	B
*ACBD5*	NM_145698.5: c.382G>A	NP_663736.2: p.(Glu128Lys)	3.993 × 10^−6^	D	D	0.35	Disease causing	Lo	D	D	32	B
*RHO*	NM_000539.3: c.448G>A	NP_000530.1: p.(Glu150Lys)	4.772 × 10^−5^	D (0.002)	D	0.71	Disease causing	Med	D	D	27.3	B
*RP1*	NM_006269.2: c.3836_3837insT	NP_006260.1: p.(Pro1282SerfsTer2)	N/A	N/A	N/A	N/A	Disease causing	N/A	N/A	N/A	N/A	B
*PROM1*	NM_006017.3: c.1919C>T	NP_006008.1: p.(Ser649Leu)	2.898 × 10^−5^	T	B	0.006	Disease causing	Neutral	D	D	14.69	B

AF: allele frequency, N/A: not available, D: damaging, T: tolerated, and B: benign. p.(?): Protein consequence unknown; used for variants (typically splice-site changes) where the effect at the protein level cannot be reliably predicted.

**Table 4:** List of all previously reported variants in non-syndromic retinitis pigmentosa from the Pakistani population.

Gene	cDNA Change	Protein Change	Variant Type	Disease	Inheritance	Reference
*ABCA4*	c.6658C>T	p.(Gln2220Ter)	Nonsense	RP	AR	[25]
*BEST1*	c.418C>G	p.(Leu140Val)	Missense	RP	AR	[26]
*CERKL*	c.316C>A	p.(Arg106Ser)	Missense	RP	AR	[27]
*CERKL*	c.847C>T	p.(Arg283Ter)	Nonsense	RP	AR	[25,28,29]
*CLRN1*	c.92C>T	p.(Pro31Leu)	Missense	RP	AR	[30]
*CLRN1*	c.461T>G	p.(Leu154Trp)	Missense	RP	AR	[30]
*CNGA1*	c.626_627del	p.(Ile209SerfsTer26)	Frameshift deletion	RP	AR	[31]
*CNGA1*	c.1298G>A	p.(Gly433Asp)	Missense	RP	AR	[32].
*CNGB1*	c.412-1G>A	p.(?)	Splice site	RP	AR	[18]
*CNGB1*	c.2284C>T	p.(Arg762Cys)	Missense	RP	AR	[18]
*CNGB1*	c.2493-2A>G	p.(?)	Splice site	RP	AR	[32]
*CNGB1*	c.852_874+25del	p.(Ile286AspfsTer9)	Frameshift deletion	RP	AR	[33]
*CNGB1*	c.2497_2498delAT	p.(Ile833SerfsTer18)	Frameshift deletion	RP	AR	[34]
*CRB1*	c.2234C>T	p.(Thr745Met)	Missense	RP	AR	[32]
*CRB1*	c.2536G>A	p.(Gly846Arg)	Missense	RP	AR	[35]
*CRB1*	c.3296C>A	p.(Thr1099Lys)	Missense	RP	AR	[18]
*CRB1*	c.3343_3352del	p.(Gly1115IlefsTer23)	Frameshift deletion	RP	AR	[36]
*CRB1*	c.3347T>C	p.(Leu1071Pro)	Missense	RP	AR	[35]
*CRB1*	c.3962G>C	p.(Cys1321Ser)	Missense	RP	AR	[36]
*CRB1*	c.3735delA	p.(Gly1246GlufsTer36)	Frameshift deletion			[33]
*EYS*	c.8299G>T	p.(Asp2767Tyr)	Missense	RP	AR	[37]
*EYS*	c.5571_5576delinsCTAGAT	p.(Leu1858Ter)	Nonsense	RP	AR	[15]
*EYS*	c.6137G>A	p.(Trp2046Ter)	Nonsense	RP	AR	[38]
*IMPG2*	c.1680T>A	p.(Tyr560Ter)	Nonsense	RP	AR	[39]
*MERTK*		p.(Arg466Lys)	Missense	RP	AR	[40]
*MERTK*		p.(Ile587Val)	Missense	RP	AR	[40]
*MERTK*		p.(Ser627Ser)	Synonymous	RP	AR	[40]
*MERTK*	IVS6-46G→A	N/A	Intronic	RP	AR	[40]
*MERTK*	IVS15-11C→A	N/A	Intronic	RP	AR	[40]
*MERTK*	c.2194C>T	p.(Arg732Ter)	Nonsense	RP	AR	[11]
*MERTK*	c.718G>T	p.(Glu240Ter)	Nonsense	RP	AR	[40]
*MERTK*	c.436C>T	p.(Gln146Ter)	Nonsense	RP	AR	[24]
*MERTK*	c.1843A>T	p.(Lys615Ter)	Nonsense	RP	AR	[41]
*PDE6A*	c. 304C>A	p.(Arg102Ser)	Missense	RP	AR	[42]
*PDE6A*	c.1408-2A>G	Splice defect	Splice site	RP	AR	[43]
*PDE6A*	c.769C>T	p.(Arg257Ter)	Nonsense	RP	AR	[44]
*PDE6A*	c.889C>T	p.(Arg256Ter)	Nonsense	RP	AR	[45]
*PDE6A*	c.1264-2A>G	p.(?)	Splice site	RP	AR	[46]
*PDE6A*	c.1630C>T	p.(Arg544Trp)	Missense	RP	AR	[25]
*PDE6A*	c.2218_2219insT	p.(ALA740ValfsTer2)	Frameshift insertion	RP	AR	[46]
*PDE6B*	c.1160C>T	p.(Pro387Leu)	Missense	RP	AR	[47]
*PDE6B*	c.1655G>A	p.(Arg552Gln)	Missense	RP	AR	[47]
*PDE6B*	c.1722+1G>A	p.(?)	Splice site	RP	AR	[18]
*PDE6B*	c.12_15delTGAG	p.(Ser4ArgfsTer23)	Frameshift deletion	RP	AR	[44]
*PDE6B*	c.427del	p.(Ala143LeufsTer7)	Frameshift deletion	RP	AR	[48]
*PDE6B*	c.243delG	p.(Arg82AlafsTer68)	Frameshift deletion	RP	AR	[44]
*PDE6B*	c.938C>T	p.(Thr313Ile)	Missense	RP	AR	[49]
*PDE6B*	c.1921-20_1921-3del	Affect mRNA splicing	Splice site	RP	AR	[50]
*PDE6C*	c.480delG	p.(Asn161ThrfsTer33)	Frameshift deletion	RP	AR	[11]
*PROM1*	c.1726C>T	p.(Gln576Ter)	Nonsense	RP	AR	[51]
*PROM1*	c.1946C>T	p.(Ser649Leu)	Missense	RP	AR	[38]
*PROM1*	c.1649C>G	p.(Ser550Ter)	Nonsense	RP	AR	[24]
*RHO*	c.448G>A	p.(Glu150Lys)	Missense	RP	AR	[11,52]
*RHO*	c.1045T>G	p.(*349GluextTer52)	Stop loss	RP	AR	[53]
*RP1*	c.1458_1461dup	p.(Glu488Ter)	Nonsense	RP	AR	[45,54]
*RP1*	c.4555del	p.(Arg1519GlufsTer2)	Frameshift deletion	RP	AR	[45]
*RP1*	c.5252del	p.(Asn1751IlefsTer4)	Frameshift deletion	RP	AR	[45]
*RP1*	c.615+1G>A	p.(?)	Splice site	RP	AR	[13]
*RP1*	c.4703delA	p.(Arg1519GlufsTer2)	Frameshift deletion	RP	AR	[45]
*RP1*	c.1461-1465insTGAA	truncated protein	Frameshift deletion	RP	AR	[45]
*RP1*	c.5400delA	p.(Asp1751fsTer1754)	Frameshift deletion	RP	AR	[45]
*RP1*	c.1606insTGAA	p.(Glu488Ter)	Nonsense	RP	AR	[45]
*RP1*	11,117 bp deletion	deletion of 3 exons	Large deletion	RP	AR	[55]
*RP1*	c.551_552dupTA	p.(Gln185TyrfsTer4)	Frameshift duplication	RP	AR	[55]
*RP1*	c.787+1G>A	p.(Iso263Aspfs8Ter)	Splice site	RP	AR	[55]
*RP1*	c.3697delT	p.(Ser1233ProfsTer22)	Frameshift deletion	RP	AR	[55]
*RP1*	c.1126C>T	p.(Arg376Ter)	Nonsense	RP	AR	[38]
*RP1*	c.6098G>A	p.(Cys2033Tyr)	Missense	RP	AR	[38]
*RGPR*	c.2426_2427del	p.(Glu809GlyfsTer25)	Frameshift deletion	RP	XL	[32,56]
*SEMA4A ‡*	c.1033G>C	p.(Asp345His)	Missense	RP	AR	[57]
*SEMA4A ‡*	c.1049T>G	p.(Phe350Cys)	Missense	RP	AR	[57]
*SEMA4A ‡*	c.2138G>A	p.(Arg713Gln)	Missense	RP	AD	[32]
*RP1*	c.1118C>T	p.(Thr373Ile)	Missense	RP	AR	[32]
*TTC8*	c.115-2A>G	p.(?)	Splice site	RP	AR	[58]
*TTC8*	c.768+5G>A	p.(?)	Splice site	RP	AR	[33]
*TULP1*	c.1138A>G	p.(Thr380Ala)	Missense	RP	AR	[22,59]
*TULP1*	c.1445G>A	p.(Arg482Gln)	Missense	RP	AR	[59]
*TULP1*	c.1466A>G	p.(Lys489Arg)	Missense	RP	AR	[22,60]
*TULP1*	c.1495+4A>C	p.(P499RfsTer104)	Splice site	RP	AR	[44]
*TULP1*	c.1561C>T	p.(Pro521Ser)	Missense	RP	AR	[44]
*TULP1*	c.286_287delGA	p.(Glu96GlyfsTer77)	Frameshift deletion	RP	AR	[44]
*TULP1*	c.238C>T	p.(Gln80Ter)	Nonsense	RP	AR	[11]
*TULP1*	c.1307A>G	p.(Lys436Arg)	Missense	RP	AR	[48]
*TULP1*	c.1274T>C	p.(Ile425Thr)	Missense	RP	AR	[61]
*TULP1*	c.855dupC	p.(Val286ArgfsTer98)	Frameshift duplication	RP	AR	[33]
*TULP1*	c.1444C>T	p.(Arg482Trp)	Missense	AR	RP	[24]
*ZNF513*	c.1015T>C	p.(Cys339Arg)	Missense	RP	AR	[62,63]
*C8orf37*	c.244-2A>C	truncated protein	Splice site	RP	AR	[64]
*C8orf37*	c.555G>A	p.(Trp185Ter)	Nonsense	RP	AR	[64]
*FAM161A*	c.1309A>T	p.(Arg437Ter)	Nonsense	RP	AR	[65]
*FAM161A*	c.1139G>T	p.(Arg380Leu)	Missense	RP	AR	[38]
*IMPDH1*	c.676G>A	p.(Asp226Asn)	Missense	RP	AR	[66]
*LRAT*	c.196G>C	p.(Gly66Arg)	Missense	RP	AR	[33,67]
*LRAT*	c.418G>T	p.(Glu140Ter)	Nonsense	RP	AR	[38]
*LRAT*	c.538A>T	p.(Lys180Ter)	Nonsense	RP	AR	[38]
*LRAT*	c.541-15T>G	p.(?)	Splice site	RP	AR	[68]
*PLRG1*	c.466A>T	p.(Ile165Phe)	Missense	RP	AR	[67]
*RPE65*	c.361del T	p.(S121LfsTer6)	Frameshift deletion	RP	AR	[60,69]
*RPE65*	c.131G>A	p.(Arg44Gln)	Missense	RP	AR	[60]
*RPE65*	c.550G>T	p.(Glu184Ter)	Nonsense	RP	AR	[48]
*RPE65*	c.351delC	p.(Arg118GlyfsTer9)	Frameshift deletion	RP	AR	[61]
*IFT140*	c.998G>A	p.(Cys333Tyr)	Missense	RP	AR	[70]
*IFT140*	c.1451C>T	p.(Thr484Met)	Missense	RP	AR	[70]
*NR2E3*	c.227G>A	p.(Arg76Gln)	Missense	RP	AR	[38]
*RBP3*	c.3353_3354delCT	p.(Ser1118CysfsTer3)	Frameshift deletion	RP	AR	[38]
*CLCC1*	c.75C>A	p.(Asp25Glu)	Missense	RP	AR	[71]
*DHX38*	c.971G>A	p.(Arg324Gln)	Missense	RP	AR	[72]
*RDH5*	c.602C>T	p.(Ser201Phe)	Missense	RP	AR	[73]
*PRPF3*	c.1481C>T	p.(Thr494Met)	Missense	RP	AR	[34]
*SNRNP200*	c.3269G>A	p.(Arg1090Gln)	Missense	RP	AD	[74]
*HGSNAT*	c.1843G>A	p.(Ala615Thr)	Missense	RP	XL	[24]

* AR: autosomal recessive, AD: autosomal dominant, XL: X-lined, and RP: retinitis pigmentosa. ‡ Only RP-associated SEMA4A variants/studies were included.

## 4. Discussion

In this study, the targeted panel sequencing of 16 consanguineous Pakistani families segregating the non-syndromic retinitis pigmentosa phenotype revealed 15 distinct variants across 14 genes. This study capitalizes on the high homozygosity in inbred families, a feature particularly pronounced in Pakistan, where consanguinity contributes to an elevated RP prevalence (1:1000–2000). Notably, our findings expand the RP mutational landscape with four novel variants. Among these, two novel recessively segregating variants including p.(Val220Met) and p.(Pro1282SerfsTer2) were identified in *RP1*. *RP1* exhibits a well-recognized position-dependent allelic architecture. Most autosomal dominant *RP1* disease is caused by truncating variants within the exon 4 “hotspot” (~codons 500–1053), which can escape nonsense-mediated decay and exert a dominant-negative effect [75]. In contrast, biallelic *RP1* variants, including early/N-terminal truncations, are documented to cause autosomal recessive RP, with heterozygous carriers often unaffected, as shown by the homozygous p.(Arg338Ter) mutation reported in an Indonesian family [76]. Consistent with these findings, in family RP099, we identified a novel homozygous *RP1* missense variant p.(Val220Met) located within the N-terminal DCX2 domain (~residues 150–228) of the RP1 protein. This variant lies outside the well-established RP1 dominant truncation hotspot and is, therefore, segregating with recessive RP1-associated rod–cone degeneration rather than a dominant disease mechanism. The DCX domains are known to be involved in microtubule-associated functions important for photoreceptor structure and maintenance [77]. Missense variants in similar regions have been associated with disruption of these functions in experimental systems [78]. In contrast to truncating *RP1* mutations that typically cause severe RP, this hypomorphic missense variant may result in a milder phenotype, consistent with previous reports describing Pakistani *RP1*-linked families [13]. *RP1* biallelic variants, including frameshifts, have also been documented in non-Pakistani cohorts with autosomal recessive RP, often presenting with early-onset symptoms [79]. Similarly, family RP206 harbored a novel *RP1* frameshift variant, p.(Pro1282SerfsTer2), which introduces a premature stop codon two residues downstream and truncates the protein distal to the canonical dominant hotspot in exon 4. This variant is predicted to truncate the C-terminal region of the protein, resulting in loss of approximately 40.5% of the protein. Given its position and the established role of loss-of-function variants in recessive RP1-associated disease, this variant is best interpreted as a recessive loss-of-function allele rather than a dominant-negative mutation. Comparable distal truncating RP1 variants [55,76] have been reported to act as null alleles and contribute to autosomal recessive retinal degenerations. This variant is also predicted to cause ~40.5% loss of the functional protein (calculated using Mutalyzer; https://mutalyzer.nl/ (accessed on 15 January 2026)), the C-terminal domain required for axonemal anchoring, disc stacking, and higher-order microtubule organization, resulting in substantial loss of RP1 function [45,80].

In family RP162, a novel splice-site variant in *PDE6B* (c.2021+5G>A), located in intron 15, was predicted to affect normal splicing of exon 16. Such aberrant splicing is predicted to result in aberrant transcripts, potentially leading to frameshift and premature truncation or loss of the catalytic domain of PDE6B required for cGMP hydrolysis during phototransduction, although functional validation is required to confirm the splicing impact [81]. In silico analysis using SpliceAI predicts a high delta score of 0.72 for donor site loss, indicating a strong likelihood of aberrant splicing, such as exon skipping or intron retention, which would lead to a frameshift or truncated protein lacking full catalytic activity and potentially disrupting phototransduction. Previously, an intronic deletion (NM_000283.3:c.1921-20_1921-3del) in intron 15 affecting the acceptor splice site at the 5′ end of the exon 16, segregating in a consanguineous Pakistani family with autosomal recessive RP [50], was experimentally validated via the minigene assay and the data showed that aberrant splicing resulted in an in-frame deletion of the first 18 bp of exon 16, disrupting the catalytic domain. The family segregating that variant (NM_000283.3: c.1921-20_1921-3del) had an early-onset night blindness progressing to severe vision loss, peripheral retinal degeneration with bone spicules, and attenuated vessels, clinical features broadly consistent with those observed in our family RP162, although detailed electrophysiological data are not available. Symptom onset occurred at early onset with the initial complaint of night blindness. Furthermore, our variant’s impact on the splice donor site parallels another intronic *PDE6B* mutation (c.1921-9C>G) at position −9 in intron 15, which creates a novel acceptor splice site leading to an 8 bp insertion and frameshift p.(Thr641LeufsTer5) in exon 16, as validated by the minigene assay in a cohort of Caucasus Jewish patients with arRP exhibiting childhood-onset night blindness, visual field constriction, and macular involvement [82].

Family RP165 carried a novel *PRCD* nonsense variant, p.(Ser38Ter), which introduces a premature termination codon at 38-position in full-length 54 amino acid PRCD protein. This truncation removes the C-terminal 16 amino acids (≈29.6% of the full-length protein) located downstream of the highly conserved N-terminal region, as predicted by Mutalyzer (https://mutalyzer.nl/ (accessed on 15 January 2026)). The lost C-terminal segment is important for proper membrane association, palmitoylation-dependent targeting, and regulation of photoreceptor disc morphogenesis [83] (Figure 3C). It mirrors loss-of-function mutations in canine models of PRCD, where homozygous mutations in the PRCD homolog cause autosomal recessive photoreceptor degeneration with similar structural and functional deficits in rod maintenance [84]. Comparable truncating variants in nearby or earlier positions within the conserved N-terminal region, such as the nonsense mutations c. 52C>T; p.(Arg18Ter) in a Turkish family and c.64C>T; p.(Arg22Ter) in Israeli patients, have been reported in association with autosomal recessive RP, often presenting with early-onset phenotypes. Additionally, missense variants like c.5G>A; p.(Cys2Tyr) in the same conserved region affect protein stability and localization, leading to aggregation and photoreceptor loss [84,85,86].

Our study identified several variants with established pathogenic roles in retinal degeneration previously documented in Pakistani and other populations. In families RP005 and RP172, we observed the recurrent *CERKL* nonsense variant p.(Arg257Ter) (rs121909398), previously characterized in Pakistani RP pedigrees for its disruptive effects on ceramide kinase function and photoreceptor survival [25,28,29]. This variant has also been documented globally from Spanish, Jordanian, and other cohorts with autosomal recessive RP, where it is associated with early macular involvement and functional studies confirmed its pathogenicity [87,88]. Family RP010 harbored the *SAG* nonsense variant p.(Arg292Ter), also ClinVar reported, but unreported previously from the Pakistani population. This variant has been widely reported worldwide, including in Hispanic and Japanese families with autosomal recessive RP, where it disrupts phototransduction and leads to Oguchi disease-like features along with RP progression [89,90,91]. Family RP024 was identified with *TTC8* splice-site variant c.115-2A>G (rs587777809), which has been reported previously in other Pakistani families with arRP, where it compromises ciliary protein function through aberrant splicing [58]. The *TTC8* variant appears enriched in South Asian populations, with limited global reports beyond Pakistani cohorts [92]. Family RP049 carried the *RPE65* disease-causing variant leading to p.(Arg91Trp) (rs61752871), with no previous report from the Pakistani population but well-established pathogenicity in global cohorts for autosomal recessive RP, including early-onset night blindness and reduced isomerohydrolase activity [93]. In family RP060, the *MERTK* nonsense variant p.(Gln146Ter) was detected, consistent with recent Pakistani reports [24]. Similar *MERTK* truncations have been identified in RP families, often causing phagocytosis defects and severe retinal degeneration [94,95]. Family RP076 harbored the p.(Phe229Leu) (rs752597235) mutation in *IDH3B* that is listed in variant databases for RP (https://www.ncbi.nlm.nih.gov/clinvar/variation/971237/ (accessed on 15 January 2026)), but there is no report available regarding functional evidence of this variant, suggesting a rare or understudied role in mitochondrial-dysfunction-related RP. According to ACMG/AMP guidelines, the variant satisfies criteria including PM2, PP3, and PP1; however, these represent supporting-level evidence and do not meet the threshold for pathogenic or likely pathogenic classification. The variant was, therefore, retained as VUS. The *EYS* stop–gain variant p.(Arg164Ter) (rs794727631) in family RP168 has been documented in families with severe early-onset RP, where it disrupts extracellular matrix integrity. This variant appears in global ClinVar entries, with reports from diverse populations, including Iranian cohorts [96]. Similarly, family RP170 harbored the *PDE6A* variant leading to p.(Arg257Ter), previously documented from Spanish [97] and Pakistani populations [44,46]. *PDE6A* nonsense variants like this are linked to autosomal recessive RP, with genotype–phenotype correlations showing rod–cone dystrophy in European and Asian studies [98]. Family RP174 carried the *ACBD5* missense variant causing p.(Glu128Lys). This variant is rare in population databases and is supported by computational predictions suggesting a potential deleterious effect. Segregation analysis within the family was consistent with the expected inheritance pattern. However, in the absence of functional validation and strong supportive evidence, the variant remains classified as a variant of VUS according to ACMG/AMP guidelines.

Family RP189 harbored the *RHO* missense variant p.(Glu150Lys) (rs104893791), a well-established founder mutation in the Pakistani RP cohort that exhibits recessive inheritance, contrasting with its dominant effects in European populations [11,52]. Global studies confirm its recessive pattern in South Asian families, with homozygous cases typically associated with night blindness and field constriction in reported cohorts, while heterozygous carriers may be asymptomatic. Family RP244 carried *PROM1* p.(Ser649Leu) (rs761911901), a rare missense variant listed in ClinVar with limited published evidence. This variant is associated with autosomal recessive RP in database entries, with limited global reports from Indian and European cohorts showing macular involvement [92].

The mutational spectrum observed in our Pakistani non-syndromic RP cohort shows both overlap with and divergence from patterns reported in other South Asian and global studies. Similar to previous work from Pakistan and India, *RP1, TULP1, MERTK, PDE6B*, and *CRB1/PDE6A* appear to be among the most frequently mutated genes, collectively accounting for a substantial proportion of all autosomal recessive variants [32]. This is consistent with previous reports where *RP1* and *TULP1* were particularly enriched in consanguineous families, highlighting the influence of founder effects and autozygosity in this population [11,15]. The proportion of truncating variants in our population (51.4%), particularly in *PDE6B, RP1*, and *TULP1*, appears higher than the ~35–40% typically reported in European cohorts [99]. This enrichment is likely attributable to the high consanguinity rate in the Pakistani population, which facilitates homozygosity for rare, deleterious alleles. Domain-level clustering of missense mutations in functionally critical regions, such as the DCX domain of RP1, the tubby domain of *TULP1*, and the catalytic domain of *PDE6B*, appears consistent with genotype-structure correlations described in previous studies (Figure 5) [100,101]. Interestingly, the combined contribution of *CRB1* and *PDE6A* (13%) in our cohort aligns with recent South Asian studies reporting that these loci account for over 15% of autosomal recessive RP cases [98]. In contrast, in European and East Asian cohorts, *CRB1* tends to rank lower in frequency, and *PDE6A* often contributes less than 5%, suggesting potential ethnic and geographic differences in gene prevalence [100,102].

## 5. Conclusions

In conclusion, this comprehensive genetic analysis of 16 consanguineous non-syndromic RP segregating Pakistani families reveals a heterogeneous mutational spectrum. Our identification of four novel variants in *RP1, PDE6B*, and *PRCD* adds to the expanding catalog of reported variants, particularly enriching the allelic diversity in these genes within South Asian populations. Recurrent mutations in genes like *CERKL, RHO*, and *EYS* suggest population-specific patterns that warrant further investigation. Descriptive analysis indicated missense variants as predominant in our population (Table 4). Recurrent involvement of genes such as *RP1, TULP1, MERTK*, and *PDE6B* may help prioritize these loci for targeted genetic screening and variant interpretation in Asian populations. Variant distribution across key functional domains may provide a framework for prioritizing variants in future studies and supports the design of functional assays to evaluate their biological impact. However, the absence of a large ethnically matched Pakistani control cohort represents a limitation, necessitating reliance on segregation analysis, phenotype concordance, and biological plausibility for variant interpretation rather than population frequency alone. In addition, the lack of functional validation limits definitive conclusions regarding the biological impact of identified variants. Furthermore, limited access to advanced ophthalmic diagnostic tools, such as electroretinography and high-resolution imaging, remains a common challenge in resource-limited settings, which may affect the depth of clinical characterization. These findings not only provide a descriptive overview of the genetic architecture of RP but also may help refine the diagnostic landscape for RP in high-consanguinity settings, supporting the potential utility of targeted panels in unraveling autosomal recessive etiologies. Future studies incorporating functional assays are required to validate pathogenicity of novel variants and refine genotype–phenotype correlations.

## Data Availability

The original contributions presented in this study are included in the article/Supplementary Materials. Further inquiries can be directed to the corresponding authors.

## Supplementary Materials

The following supporting information can be downloaded at: https://www.mdpi.com/article/10.3390/genes17050529/s1. Figure S1: Pedigree analysis, Sanger sequencing validation, and segregation of identified variants in 12 Pakistani families with retinitis pigmentosa. Pedigrees (A-L) represent families RP005, RP010, RP024, RP049, RP060, RP076, RP099, RP168, RP170, RP172, RP174, RP189, and RP244. Squares denote males and circles denote females; filled symbols indicate affected individuals, while open symbols represent unaffected individuals. Double lines indicate consanguineous unions. Arrows mark the probands. Genotypes are indicated as homozygous mutant (M1/M1), heterozygous (M1/+), or wild type (+/+). Representative electropherograms show Sanger sequencing validation of identified variants in affected and unaffected family members, with variant positions highlighted by red boxes. The corresponding gene and nucleotide changes are indicated for each family, including *CERKL* (c.769C>T), *SAG* (c.874C>T), *TTC8* (c.115-2A>G), *RPE65* (c.271C>T), *MERTK* (c.436C>T), *IDH3B* (c.685T>C), *EYS* (c.490C>T), *PDE6A* (c.769C>T), *ACBD5* (c.382G>A), *RHO* (c.448G>A), and *PROM1* (c.1919C>T). The segregation patterns confirm co-segregation of the identified variants with the disease phenotype within each family. Table S1: List of 344 candidate genes screened in panel sequencing.

## Abbreviations

The following abbreviations are used in this manuscript:

ACMG	American College of Medical Genetics and Genomics
ADRP	Autosomal Dominant Retinitis Pigmentosa
ARRP	Autosomal Recessive Retinitis Pigmentosa
BWA-MEM	Burrows–Wheeler Aligner—MEM Algorithm
ERG	Electroretinography
EOSRD	Early-Onset Severe Retinal Dystrophy
GATK	Genome Analysis Toolkit
gnomAD	Genome Aggregation Database
GRCh37	Genome Reference Consortium Human Build 37
LCA	Leber Congenital Amaurosis
LOVD	Leiden Open Variation Database
MAF	Minor Allele Frequency
OCT	Optical Coherence Tomography
RP	Retinitis Pigmentosa
SVI	Sequence Variant Interpretation
XLRP	X-Linked Retinitis Pigmentosa

## References

[1] García Bohórquez B., Aller E., Rodríguez Muñoz A., Jaijo T., García G.G., Millán J.M. (2021). Updating the genetic landscape of inherited retinal dystrophies. Front. Cell Dev. Biol..

[2] Cross N., van Steen C., Zegaoui Y., Satherley A., Angelillo L. (2022). Retinitis Pigmentosa: Burden of Disease and Current Unmet Needs. Clin. Ophthalmol..

[3] Ferrari S., Di Iorio E., Barbaro V., Ponzin D., Sorrentino F.S., Parmeggiani F. (2011). Retinitis pigmentosa: Genes and disease mechanisms. Curr. Genom..

[4] Salmaninejad A., Motaee J., Farjami M., Alimardani M., Esmaeilie A., Pasdar A. (2019). Next-generation sequencing and its application in diagnosis of retinitis pigmentosa. Ophthalmic Genet..

[5] Weisschuh N., Mazzola P., Zuleger T., Schaeferhoff K., Kühlewein L., Kortüm F., Witt D., Liebmann A., Falb R., Pohl L. (2024). Diagnostic genome sequencing improves diagnostic yield: A prospective single-centre study in 1000 patients with inherited eye diseases. J. Med. Genet..

[6] Daiger S.P., Sullivan L.S., Bowne S.J. (2013). Genes and mutations causing retinitis pigmentosa. Clin. Genet..

[7] O’Neal T.B., Tripathy K., Luther E.E. (2025). Retinitis Pigmentosa. StatPearls [Internet].

[8] Jiman O.A., Taylor R.L., Lenassi E., Smith J.C., Douzgou S., Ellingford J.M., Barton S., Hardcastle C., Fletcher T., Campbell C. (2020). Diagnostic yield of panel-based genetic testing in syndromic inherited retinal disease. Eur. J. Hum. Genet..

[9] Britten-Jones A.C., Gocuk S.A., Goh K.L., Huq A., Edwards T.L., Ayton L.N. (2023). The Diagnostic Yield of Next Generation Sequencing in Inherited Retinal Diseases: A Systematic Review and Meta-analysis. Am. J. Ophthalmol..

[10] Wawrocka A., Walczak-Sztulpa J., Kuszel L., Niedziela-Schwartz Z., Skorczyk-Werner A., Bernardczyk-Meller J., Krawczynski M.R. (2024). Coexistence of Retinitis Pigmentosa and Ataxia in Patients with PHARC, PCARP, and Oliver–McFarlane Syndromes. Int. J. Mol. Sci..

[11] Marwan M., Dawood M., Ullah M., Shah I.U., Khan N., Hassan M.T., Karam M., Rawlins L.E., Baple E.L., Crosby A.H. (2023). Unravelling the genetic basis of retinal dystrophies in Pakistani consanguineous families. BMC Ophthalmol..

[12] Zafar S., Ahmed K., Ali A., Baig R. (2017). Retinitis pigmentosa genes implicated in South Asian populations: A systematic review. J. Pak. Med. Assoc..

[13] Rashid A., Munir A., Zahid M., Ullah M., Rehman A.U. (2025). Exome sequencing identifies a homozygous splice site variant in RP1 as the underlying cause of autosomal recessive retinitis pigmentosa in a Pakistani family. Ann. Med..

[14] Ullah M., Rehman A.U., Quinodoz M., Rashid A., Cancellieri F., Munir A., Kaminska K., Iqbal A., Javed S., Dawood M. (2025). A comprehensive genetic landscape of inherited retinal diseases in a large Pakistani cohort. npj Genom. Med..

[15] Tehreem R., Chen I., Shah M.R., Li Y., Khan M.A., Afshan K., Chen R., Firasat S. (2022). Exome sequencing identified molecular determinants of retinal dystrophies in nine consanguineous Pakistani families. Genes.

[16] Pei X.M., Yeung M.H.Y., Wong A.N.N., Tsang H.F., Yu A.C.S., Yim A.K.Y., Wong S.C.C. (2023). Targeted sequencing approach and its clinical applications for the molecular diagnosis of human diseases. Cells.

[17] Bhérer C., Eveleigh R., Trajanoska K., St-Cyr J., Paccard A., Nadukkalam Ravindran P., Caron E., Bader Asbah N., McClelland P., Wei C. (2024). A cost-effective sequencing method for genetic studies combining high-depth whole exome and low-depth whole genome. npj Genom. Med..

[18] Azam M., Collin R.W.J., Malik A., Khan M.I., Shah S.T.A., Shah A.A., Hussain A., Sadeque A., Arimadyo K., Ajmal M. (2011). Identification of Novel Mutations in Pakistani Families With Autosomal Recessive Retinitis Pigmentosa. Arch. Ophthalmol..

[19] Lieviant J.A., Chan C.M., Bylstra Y., Jain K., Teo J.X., Lim W.W., Kam S., Chao T.W., Chai Bin Siew N., Davila S. (2025). Determinants of diagnostic yield in a multi-ethnic Asian inherited retinal disease cohort. Eur. J. Hum. Genet..

[20] Wawrocka A., Socha M., Walczak-Sztulpa J., Koczyk G., Skorczyk-Werner A., Krawczyński M.R. (2023). Molecular Re-Diagnosis with Whole-Exome Sequencing Increases the Diagnostic Yield in Patients with Non-Syndromic Retinitis Pigmentosa. Diagnostics.

[21] Najmabadi H., Hu H., Garshasbi M., Zemojtel T., Abedini S.S., Chen W., Hosseini M., Behjati F., Haas S., Jamali P. (2011). Deep sequencing reveals 50 novel genes for recessive cognitive disorders. Nature.

[22] Iqbal M., Naeem M.A., Riazuddin S.A., Ali S., Farooq T., Qazi Z.A., Khan S.N., Husnain T., Riazuddin S., Sieving P.A. (2011). Association of pathogenic mutations in TULP1 with retinitis pigmentosa in consanguineous Pakistani families. Arch. Ophthalmol..

[23] de Bruijn S.E., Fadaie Z., Cremers F.P., Kremer H., Roosing S. (2021). The impact of modern technologies on molecular diagnostic success rates, with a focus on inherited retinal dystrophy and hearing loss. Int. J. Mol. Sci..

[24] Basharat R., de Bruijn S.E., Zahid M., Rodenburg K., Hitti-Malin R.J., Rodríguez-Hidalgo M., Boonen E.G., Jarral A., Mahmood A., Corominas J. (2024). Next-generation sequencing to genetically diagnose a diverse range of inherited eye disorders in 15 consanguineous families from Pakistan. Exp. Eye Res..

[25] Hussain A., Shahzad A., Venselaar H., Bokhari H., de Wijs I.J., Hoefsloot L.H., Gill M., Waheed N.K., Collin R.W. (2013). Homozygosity mapping identifies genetic defects in four consanguineous families with retinal dystrophy from Pakistan. Clin. Genet..

[26] Davidson A.E., Millar I.D., Urquhart J.E., Burgess-Mullan R., Shweikh Y., Parry N., O’Sullivan J., Maher G.J., McKibbin M., Downes S.M. (2009). Missense mutations in a retinal pigment epithelium protein, bestrophin-1, cause retinitis pigmentosa. Am. J. Hum. Genet..

[27] Ali M., Ramprasad V.L., Soumittra N., Mohamed M.D., Jafri H., Rashid Y., Danciger M., McKibbin M., Kumaramanickavel G., Inglehearn C.F. (2008). A missense mutation in the nuclear localization signal sequence of CERKL (p. R106S) causes autosomal recessive retinal degeneration. Mol. Vis..

[28] Littink K.W., Koenekoop R.K., van den Born L.I., Collin R.W., Moruz L., Veltman J.A., Roosing S., Zonneveld M.N., Omar A., Darvish M. (2010). Homozygosity mapping in patients with cone–rod dystrophy: Novel mutations and clinical characterizations. Investig. Ophthalmol. Vis. Sci..

[29] Avila-Fernandez A., Riveiro-Alvarez R., Vallespin E., Wilke R., Tapias I., Cantalapiedra D., Aguirre-Lamban J., Gimenez A., Trujillo-Tiebas M.-J., Ayuso C. (2008). CERKL mutations and associated phenotypes in seven Spanish families with autosomal recessive retinitis pigmentosa. Investig. Ophthalmol. Vis. Sci..

[30] Khan M.I., Kersten F.F., Azam M., Collin R.W., Hussain A., Shah S.T.-A., Keunen J.E., Kremer H., Cremers F.P., Qamar R. (2011). CLRN1 mutations cause nonsyndromic retinitis pigmentosa. Ophthalmology.

[31] Zhang Q., Zulfiqar F., Riazuddin S.A., Xiao X., Ahmad Z., Riazuddin S., Hejtmancik J.F. (2004). Autosomal recessive retinitis pigmentosa in a Pakistani family mapped to CNGA1 with identification of a novel mutation. Mol. Vis..

[32] Khan M.I., Azam M., Ajmal M., Collin R.W., Den Hollander A.I., Cremers F.P., Qamar R. (2014). The molecular basis of retinal dystrophies in Pakistan. Genes.

[33] D’Atri I. (2020). Defining the Genetic and Molecular Basis of Inherited Eye Diseases Present in Pakistan. Ph.D. Thesis.

[34] Biswas P., Villanueva A.L., Soto-Hermida A., Duncan J.L., Matsui H., Borooah S., Kurmanov B., Richard G., Khan S.Y., Branham K. (2021). Deciphering the genetic architecture and ethnographic distribution of IRD in three ethnic populations by whole genome sequence analysis. PLoS Genet..

[35] Khaliq S., Abid A., Hameed A., Anwar K., Mohyuddin A., Azmat Z., Shami S., Ismail M., Mehdi S.Q. (2003). Mutation screening of Pakistani families with congenital eye disorders. Exp. Eye Res..

[36] Lotery A.J., Malik A., Shami S., Sindhi M., Chohan B., Maqbool C., Moore P.A., Denton M.J., Stone E.M. (2001). CRB1 mutations may result in retinitis pigmentosa without para-arteriolar RPE preservation. Ophthalmic Genet..

[37] Khan M.I., Collin R.W., Arimadyo K., Micheal S., Azam M., Qureshi N., Faradz S.M., den Hollander A.I., Qamar R., Cremers F.P. (2010). Missense mutations at homologous positions in the fourth and fifth laminin A G-like domains of eyes shut homolog cause autosomal recessive retinitis pigmentosa. Mol. Vis..

[38] Li L., Chen Y., Jiao X., Jin C., Jiang D., Tanwar M., Ma Z., Huang L., Ma X., Sun W. (2017). Homozygosity mapping and genetic analysis of autosomal recessive retinal dystrophies in 144 consanguineous Pakistani families. Investig. Ophthalmol. Vis. Sci..

[39] Bandah-Rozenfeld D., Collin R.W., Banin E., Van Den Born L.I., Coene K.L., Siemiatkowska A.M., Zelinger L., Khan M.I., Lefeber D.J., Erdinest I. (2010). Mutations in IMPG2, encoding interphotoreceptor matrix proteoglycan 2, cause autosomal-recessive retinitis pigmentosa. Am. J. Hum. Genet..

[40] Shahzadi A., Riazuddin S.A., Ali S., Li D., Khan S.N., Husnain T., Akram J., Sieving P.A., Hejtmancik J.F., Riazuddin S. (2010). Nonsense mutation in MERTK causes autosomal recessive retinitis pigmentosa in a consanguineous Pakistani family. Br. J. Ophthalmol..

[41] Rashid M., Qasim M., Ishaq R., Bukhari S.A., Sajid Z., Ashfaq U.A., Haque A., Ahmed Z.M. (2020). Pathogenic variants of AIPL1, MERTK, GUCY2D, and FOXE3 in Pakistani families with clinically heterogeneous eye diseases. PLoS ONE.

[42] Khan A.A., Waryah Y.M., Iqbal M., Baig H.M.A., Rafique M., Waryah A.M. (2021). P. arg102ser is a common Pde6α mutation causing autosomal recessive retinitis pigmentosa in Pakistani families. J. Pak. Med. Assoc..

[43] Saqib M.A.N., Nikopoulos K., Ullah E., Sher Khan F., Iqbal J., Bibi R., Jarral A., Sajid S., Nishiguchi K.M., Venturini G. (2015). Homozygosity mapping reveals novel and known mutations in Pakistani families with inherited retinal dystrophies. Sci. Rep..

[44] Ullah I., Kabir F., Gottsch C.B.S., Naeem M.A., Guru A.A., Ayyagari R., Khan S.N., Riazuddin S., Akram J., Riazuddin S. (2016). Mutations in phosphodiesterase 6 identified in familial cases of retinitis pigmentosa. Hum. Genome Var..

[45] Riazuddin S.A., Zulfiqar F., Zhang Q., Sergeev Y.V., Qazi Z.A., Husnain T., Caruso R., Riazuddin S., Sieving P.A., Hejtmancik J.F. (2005). Autosomal recessive retinitis pigmentosa is associated with mutations in RP1 in three consanguineous Pakistani families. Investig. Ophthalmol. Vis. Sci..

[46] Riazuddin S.A., Zulfiqar F., Zhang Q., Yao W., Li S., Jiao X., Shahzadi A., Amer M., Iqbal M., Hussnain T. (2006). Mutations in the gene encoding the alpha-subunit of rod phosphodiesterase in consanguineous Pakistani families. Mol. Vis..

[47] Ali S., Riazuddin S.A., Shahzadi A., Nasir I.A., Khan S.N., Husnain T., Akram J., Sieving P.A., Hejtmancik J.F., Riazuddin S. (2011). Mutations in the β-subunit of rod phosphodiesterase identified in consanguineous Pakistani families with autosomal recessive retinitis pigmentosa. Mol. Vis..

[48] Ur Rehman A., Peter V.G., Quinodoz M., Rashid A., Khan S.A., Superti-Furga A., Rivolta C. (2019). Exploring the genetic landscape of retinal diseases in North-Western Pakistan reveals a high degree of autozygosity and a prevalent founder mutation in ABCA4. Genes.

[49] Aziz N., Ullah M., Rashid A., Hussain Z., Shah K., Awan A., Khan M., Ullah I., Rehman A.U. (2023). A novel homozygous missense substitution p. Thr313Ile in the PDE6B gene underlies autosomal recessive retinitis pigmentosa in a consanguineous Pakistani family. BMC Ophthalmol..

[50] Ullah M., Rehman A.U., Folcher M., Ullah A., Usman F., Rashid A., Khan B., Quinodoz M., Ansar M., Rivolta C. (2023). A novel intronic deletion in PDE6B causes autosomal recessive retinitis pigmentosa by interfering with RNA splicing. Ophthalmic Res..

[51] Zhang Q., Zulfiqar F., Xiao X., Riazuddin S.A., Ahmad Z., Caruso R., MacDonald I., Sieving P., Riazuddin S., Hejtmancik J.F. (2007). Severe retinitis pigmentosa mapped to 4p15 and associated with a novel mutation in the PROM1 gene. Hum. Genet..

[52] Azam M., Khan M.I., Gal A., Hussain A., Shah S.T.A., Khan M.S., Sadeque A., Bokhari H., Collin R.W., Orth U. (2009). A homozygous p. Glu150Lys mutation in the opsin gene of two Pakistani families with autosomal recessive retinitis pigmentosa. Mol. Vis..

[53] Bessant D.A., Khaliq S., Hameed A., Anwar K., Payne A.M., Mehdi S.Q., Bhattacharya S.S. (1999). Severe autosomal dominant retinitis pigmentosa caused by a novel rhodopsin mutation (Ter349Glu). Hum. Mutat..

[54] Khaliq S., Abid A., Ismail M., Hameed A., Mohyuddin A., Lall P., Aziz A., Anwar K., Mehdi S. (2005). Novel association of RP1 gene mutations with autosomal recessive retinitis pigmentosa. J. Med. Genet..

[55] Kabir F., Ullah I., Ali S., Gottsch A.D., Naeem M.A., Assir M.Z., Khan S.N., Akram J., Riazuddin S., Ayyagari R. (2016). Loss of function mutations in RP1 are responsible for retinitis pigmentosa in consanguineous familial cases. Mol. Vis..

[56] Vervoort R., Lennon A., Bird A.C., Tulloch B., Axton R., Miano M.G., Meindl A., Meitinger T., Ciccodicola A., Wright A.F. (2000). Mutational hot spot within a new RPGR exon in X-linked retinitis pigmentosa. Nat. Genet..

[57] Abid A., Ismail M., Mehdi S.Q., Khaliq S. (2006). Identification of novel mutations in the SEMA4A gene associated with retinal degenerative diseases. J. Med. Genet..

[58] Riazuddin S.A., Iqbal M., Wang Y., Masuda T., Chen Y., Bowne S., Sullivan L.S., Waseem N.H., Bhattacharya S., Daiger S.P. (2010). A splice-site mutation in a retina-specific exon of BBS8 causes nonsyndromic retinitis pigmentosa. Am. J. Hum. Genet..

[59] Ajmal M., Khan M.I., Micheal S., Ahmed W., Shah A., Venselaar H., Bokhari H., Azam A., Waheed N.K., Collin R.W. (2012). Identification of recurrent and novel mutations in TULP1 in Pakistani families with early-onset retinitis pigmentosa. Mol. Vis..

[60] Maria M., Ajmal M., Azam M., Waheed N.K., Siddiqui S.N., Mustafa B., Ayub H., Ali L., Ahmad S., Micheal S. (2015). Homozygosity mapping and targeted sanger sequencing reveal genetic defects underlying inherited retinal disease in families from pakistan. PLoS ONE.

[61] Khan A., Bai X., Umair M., Han S., Habulieti X., Wang R., Zhang X. (2020). Novel homozygous TULP1 and RPE65 variants underlies recessive Retinitis pigmentosa. Preprint.

[62] Li L., Nakaya N., Chavali V.R., Ma Z., Jiao X., Sieving P.A., Riazuddin S., Tomarev S.I., Ayyagari R., Riazuddin S.A. (2010). A mutation in ZNF513, a putative regulator of photoreceptor development, causes autosomal-recessive retinitis pigmentosa. Am. J. Hum. Genet..

[63] Naz S., Riazuddin S.A., Li L., Shahid M., Kousar S., Sieving P.A., Hejtmancik J.F., Riazuddin S. (2010). A novel locus for autosomal recessive retinitis pigmentosa in a consanguineous Pakistani family maps to chromosome 2p. Am. J. Ophthalmol..

[64] Ravesh Z., El Asrag M.E., Weisschuh N., McKibbin M., Reuter P., Watson C.M., Baumann B., Poulter J.A., Sajid S., Panagiotou E.S. (2015). Novel C8orf37 mutations cause retinitis pigmentosa in consanguineous families of Pakistani origin. Mol. Vis..

[65] Rose A., Sergouniotis P., Alfano G., Muspratt-Tucker N., Barton S., Moore A., Black G., Bhattacharya S., Webster A. (2015). Diverse clinical phenotypes associated with a nonsense mutation in FAM161A. Eye.

[66] Ali S., Khan S.Y., Naeem M.A., Khan S.N., Husnain T., Riazuddin S., Ayyagari R., Hejtmancik J.F., Riazuddin S.A. (2015). Phenotypic variability associated with the D226N allele of IMPDH1. Ophthalmology.

[67] Jelani M., Jeon M., Rahman O.U., Rahim F., Naeem M., Kang C. (2015). Whole-exome sequencing identifies a novel LRAT mutation underlying retinitis punctata albescens in a consanguineous Pakistani family. Genes Genom..

[68] Chen Y., Huang L., Jiao X., Riazuddin S., Riazuddin S.A., Fielding Hetmancik J. (2018). A novel LRAT mutation affecting splicing in a family with early onset retinitis pigmentosa. Hum. Genom..

[69] Kabir F., Naz S., Riazuddin S.A., Naeem M.A., Khan S.N., Husnain T., Akram J., Sieving P.A., Hejtmancik J.F., Riazuddin S. (2013). Novel mutations in RPE65 identified in consanguineous Pakistani families with retinal dystrophy. Mol. Vis..

[70] Hull S., Owen N., Islam F., Tracey-White D., Plagnol V., Holder G.E., Michaelides M., Carss K., Raymond F.L., Rozet J.-M. (2016). Nonsyndromic retinal dystrophy due to bi-allelic mutations in the ciliary transport gene IFT140. Investig. Ophthalmol. Vis. Sci..

[71] Li L., Jiao X., D’Atri I., Ono F., Nelson R., Chan C.-C., Nakaya N., Ma Z., Ma Y., Cai X. (2018). Mutation in the intracellular chloride channel CLCC1 associated with autosomal recessive retinitis pigmentosa. PLoS Genet..

[72] Latif Z., Chakchouk I., Schrauwen I., Lee K., Santos-Cortez R.L.P., Abbe I., Acharya A., Jarral A., Ali I., Ullah E. (2018). Confirmation of the role of DHX38 in the etiology of early-onset retinitis pigmentosa. Investig. Ophthalmol. Vis. Sci..

[73] Sultan N., Ali I., Bukhari S.A., Baig S.M., Asif M., Qasim M., Naseer M.I., Rasool M. (2018). A novel mutation in RDH5 gene causes retinitis pigmentosa in consanguineous Pakistani family. Genes Genom..

[74] Astuti G.D., van Den Born L.I., Khan M.I., Hamel C.P., Bocquet B., Manes G., Quinodoz M., Ali M., Toomes C., McKibbin M. (2018). Identification of inherited retinal disease-associated genetic variants in 11 candidate genes. Genes.

[75] Foa N., Pfau M., Ansari G., Cancian G., Grimaldi G., Koller S., Berger W., Escher P., Janeschitz-Kriegl L., Rivolta C. (2025). Autosomal Dominant RP1 c.2613dupA (p.Arg872Thrfs*2) Variant Retinitis Pigmentosa Shows Linear Loss of the Ellipsoid Zone over Time with Highly Variable Phenotype. Ophthalmologica.

[76] Siemiatkowska A.M., Astuti G.D., Arimadyo K., den Hollander A.I., Faradz S.M., Cremers F.P., Collin R.W. (2012). Identification of a novel nonsense mutation in RP1 that causes autosomal recessive retinitis pigmentosa in an Indonesian family. Mol. Vis..

[77] Song D., Grieco S., Li Y., Hunter A., Chu S., Zhao L., Song Y., DeAngelis R.A., Shi L.-Y., Liu Q. (2014). A murine RP1 missense mutation causes protein mislocalization and slowly progressive photoreceptor degeneration. Am. J. Pathol..

[78] Riera M., Abad-Morales V., Navarro R., Ruiz-Nogales S., Méndez-Vendrell P., Corcostegui B., Pomares E. (2020). Expanding the retinal phenotype of RP1: From retinitis pigmentosa to a novel and singular macular dystrophy. Br. J. Ophthalmol..

[79] Silva R.S., Salles M.V., Motta F.L., Sallum J.M.F. (2020). Retinitis Pigmentosa Due to Rp1 Biallelic Variants. Sci. Rep..

[80] Salles M.V., Motta F., Sallum J.M. (2018). Retinitis Pigmentosa due to RP1 variants, phenotype and genotype aspects. Investig. Ophthalmol. Vis. Sci..

[81] Kuehlewein L., Zobor D., Stingl K., Kempf M., Nasser F., Bernd A., Biskup S., Cremers F.P., Khan M.I., Mazzola P. (2021). Clinical phenotype of PDE6B-associated retinitis pigmentosa. Int. J. Mol. Sci..

[82] Tatour Y., Tamaiev J., Shamaly S., Colombo R., Bril E., Rabinowitz T., Yaakobi A., Mezer E., Leibu R., Tiosano B. (2019). A novel intronic mutation of PDE6B is a major cause of autosomal recessive retinitis pigmentosa among Caucasus Jews. Mol. Vis..

[83] Spencer W.J., Ding J.-D., Lewis T.R., Yu C., Phan S., Pearring J.N., Kim K.-Y., Thor A., Mathew R., Kalnitsky J. (2019). PRCD is essential for high-fidelity photoreceptor disc formation. Proc. Natl. Acad. Sci. USA.

[84] Zangerl B., Goldstein O., Philp A.R., Lindauer S.J., Pearce-Kelling S.E., Mullins R.F., Graphodatsky A.S., Ripoll D., Felix J.S., Stone E.M. (2006). Identical mutation in a novel retinal gene causes progressive rod–cone degeneration in dogs and retinitis pigmentosa in humans. Genomics.

[85] Pach J., Kohl S., Gekeler F., Zobor D. (2013). Identification of a novel mutation in the PRCD gene causing autosomal recessive retinitis pigmentosa in a Turkish family. Mol. Vis..

[86] Nevet M., Shalev S., Zlotogora J., Mazzawi N., Ben-Yosef T. (2010). Identification of a prevalent founder mutation in an Israeli Muslim Arab village confirms the role of PRCD in the aetiology of retinitis pigmentosa in humans. J. Med. Genet..

[87] Barandika O., Irigoyen C., Anasagasti A., Egiguren G., Ezquerra-Inchausti M., López de Munain A., Ruiz-Ederra J. (2016). A Cost-Effective Mutation Screening Strategy for Inherited Retinal Dystrophies. Ophthalmic Res..

[88] Azab B., Dardas Z., Aburizeg D., Al-Bdour M., Abu-Ameerh M., Saleh T., Barham R., Maswadi R., Ababneh N.A., Alsalem M. (2021). Unique Variant Spectrum in a Jordanian Cohort with Inherited Retinal Dystrophies. Genes.

[89] Tawfik C.A., Elbagoury N.M., Khater N.I., Essawi M.L. (2022). Mutation analysis reveals novel and known mutations in SAG gene in first two Egyptian families with Oguchi disease. BMC Ophthalmol..

[90] Espinoza G., Brown J. (2024). S Antigen (SAG) Mutations are an important cause of retinitis pigmentosa in Texas patients of Hispanic descent. Investig. Ophthalmol. Vis. Sci..

[91] Sullivan L.S., Bowne S.J., Koboldt D.C., Cadena E.L., Heckenlively J.R., Branham K.E., Wheaton D.H., Jones K.D., Ruiz R.S., Pennesi M.E. (2017). A Novel Dominant Mutation in SAG, the Arrestin-1 Gene, Is a Common Cause of Retinitis Pigmentosa in Hispanic Families in the Southwestern United States. Investig. Ophthalmol. Vis. Sci..

[92] Sen P., Natarajan S., Maitra P., Srilekha S., Porkodi P., Harshavardhini G., Muna B., Khetan V., Mathavan S., Bhende P. (2023). Next-generation sequencing-based genetic testing and phenotype correlation in retinitis pigmentosa patients from India. Indian J. Ophthalmol..

[93] Li S., Xiao X., Yi Z., Sun W., Wang P., Zhang Q. (2020). RPE 65 mutation frequency and phenotypic variation according to exome sequencing in a tertiary centre for genetic eye diseases in China. Acta Ophthalmol..

[94] Al-Khersan H., Shah K.P., Jung S.C., Rodriguez A., Madduri R.K., Grassi M.A. (2017). A novel MERTK mutation causing retinitis pigmentosa. Graefes Arch. Clin. Exp. Ophthalmol..

[95] Evans D.R., Green J.S., Johnson G.J., Schwartzentruber J., Majewski J., Beaulieu C.L., Qin W., Marshall C.R., Paton T.A., Roslin N.M. (2017). Novel 25 kb Deletion of MERTK Causes Retinitis Pigmentosa with Severe Progression. Investig. Ophthalmol. Vis. Sci..

[96] Zamani M., Sedighzadeh S., Seifi T., Negahdari S., Zeighami J., Sedaghat A., Shariati G., Galehdari H. (2022). Whole-exome sequencing deciphers the genetic profile of visual impairments in patients from Southwest Iran. Mol. Genet. Genom..

[97] Ávila-Fernández A., Cantalapiedra D., Aller E., Vallespín E., Aguirre-Lambán J., Blanco-Kelly F., Corton M., Riveiro-Álvarez R., Allikmets R., Trujillo-Tiebas M.J. (2010). Mutation analysis of 272 Spanish families affected by autosomal recessive retinitis pigmentosa using a genotyping microarray. Mol. Vis..

[98] Dawood M., Din T., Shah I., Khan N., Jan A., Marwan M., Sultan K., Nowshid M., Tahir R., Ahmed A. (2021). Novel mutations in PDE6A and CDHR1 cause retinitis pigmentosa in Pakistani families. Int. J. Ophthalmol..

[99] Hanany M., Rivolta C., Sharon D. (2020). Worldwide carrier frequency and genetic prevalence of autosomal recessive inherited retinal diseases. Proc. Natl. Acad. Sci. USA.

[100] Bhardwaj A., Yadav A., Yadav M., Tanwar M. (2022). Genetic dissection of non-syndromic retinitis pigmentosa. Indian J. Ophthalmol..

[101] Xia C.-H., Liu H., Li M., Zhang H., Xing X., Gong X. (2023). Identification and characterization of retinitis pigmentosa in a novel mouse model caused by PDE6B-T592I. Biomedicines.

[102] Narváez O.M., Zerón H.M., Torres O., Escobar M., Trujillo-Güiza M.L. (2025). Retinitis Pigmentosa Genes Implicated in the Population of America: A Systematic Review. Acta Fac. Medicae Naissensis.

