# Supplementary material for: Expanding the Mutation Spectrum of Non-Syndromic Retinitis Pigmentosa in Consanguineous Pakistani Families: Unraveling Novel Pathogenic Variants in RP1, PDE6B, and PRCD Genes for Precision Diagnosis
Source: Genes (Basel). 2026 Apr 29;17(5):529. doi: 10.3390/genes17050529 (PMC13205176; doi:10.3390/genes17050529)
Supplement: Supplementary file 1 [file genes-17-00529-s001.zip › genes-4212453-supplementary.pdf]

Supplementary Table S1. List of 344 candidate genes screened in panel sequencing

Gene	Gene	Gene	Gene	Gene
ABCA4	*ARL6*	*BEST1*	*CDH23*	*CLN6*
ABCC6	*ASIC2*	*C12orf65*	*CDH3*	*CLN8*
ABHD12	*ASIC3*	*C1QTNF5*	*CDHR1*	*CLRN1*
ACBD5	*ATF6*	*C21orf2*	*CEP164*	*CLUAP1*
ADAM9	*ATOH7*	*C2orf71*	*CEP290*	*CNGA1*
ADAMTS18	*ATP1B2*	*C5orf42*	*CEP41*	*CNGA3*
ADGRA3	*ATXN7*	*C8orf37*	*CERKL*	*CNGB1*
ADGRV1	*BBIP1*	*CA4*	*CFH*	*CNGB3*
AGTPBP1	*BBS1*	*CABP4*	*CHM*	*CNNM4*
AHI1	*BBS10*	*CACNA1F*	*CIB2*	*COL11A1*
AIFM1	*BBS12*	*CACNA2D4*	*CISD2*	*COL2A1*
AIPL1	*BBS2*	*CAPN5*	*CLCN2*	*COL9A1*
ALMS1	*BBS4*	*CC2D2A*	*CLCN3*	*CRB1*
ARL13B	*BBS5*	*CCDC66*	*CLCN7*	*CRB2*
ARL2BP	*BBS7*	*CCL2*	*CLN3*	*CROCC*
ARL3	*BBS9*	*CCR2*	*CLN5*	*CRX*
CSPP1	*FAM161A*	*GRM6*	*INVS*	*LZTFL1*
CTSD	*FBLN5*	*GUCA1A*	*IQCB1*	*MAK*
CTSF	*FLVCR1*	*GUCA1B*	*ITM2B*	*MCOLN1*
CYP4V2	*FSCN2*	*GUCY2D*	*JAG1*	*MDM1*
DFNB31	*FZD4*	*GUCY2F*	*KCNJ13*	*MERTK*
DHDDS	*GBF1*	*HARS*	*KCNV2*	*MFN2*
DHX38	*GDF6*	*HK1*	*KIAA1549*	*MFRP*
DMD	*GJA10*	*HMCN1*	*KIF11*	*MFSD8*
DNAJC5	*GNAT1*	*IDH3B*	*KIF7*	*MITF*

Gene	Gene	Gene	Gene	Gene
DTHD1	*GNAT2*	*IFT140*	*KIZ*	*MKKS*
EFEMP1	*GNGT1*	*IFT172*	*KLHL7*	*MKS1*
ELOVL4	*GNPTAB*	*IFT27*	*LCA5*	*MPP5*
EMC1	*GNPTG*	*IMPDH1*	*LPCAT1*	*MTTP*
ERCC6	*GPR125*	*IMPG1*	*LRAT*	*MVK*
ERCC8	*GPR179*	*IMPG2*	*LRIT3*	*MYO7A*
EYS	*GRK1*	*INPP5E*	*LRP5*	*NDP*
NEK2	*NPHP1*	*NR2E1*	*NRL*	*NYX*
NEUROD1	*NPHP3*	*NR2E3*	*NXNL1*	*OAT*
NMNAT1	*NPHP4*	*NR2F1*	*NXNL2*	*OFD1*
OPA1	*PEX1*	*PITPNM3*	*RD3*	*RPGR*
OPA3	*PEX10*	*PLA2G5*	*RDH11*	*RPGRIP1*
OPN1LW	*PEX11B*	*POMGNT1*	*RDH12*	*RPGRIP1L*
OPN1MW	*PEX12*	*PPT1*	*RDH5*	*RRAS2*
OPN1SW	*PEX13*	*PRCD*	*RDH8*	*RS1*
OTX2	*PEX14*	*PRKCZ*	*REEP6*	*SAG*
PANK2	*PEX16*	*PROM1*	*RGR*	*SDCCAG8*
PAX2	*PEX19*	*PRPF3*	*RGS9*	*SEMA4A*
PAX6	*PEX2*	*PRPF31*	*RGS9BP*	*SLC24A1*
PCDH15	*PEX26*	*PRPF4*	*RHO*	*SLC38A8*
PCYT1A	*PEX3*	*PRPF6*	*RIMS1*	*SLC4A7*
PDCL	*PEX5*	*PRPF8*	*RLBP1*	*SLC6A6*
PDE6A	*PEX6*	*PRPH2*	*ROM1*	*SLC7A14*
PDE6B	*PEX7*	*RAB28*	*RP1*	*SNRNP200*
PDE6C	*PFDN5*	*RAX2*	*RP1L1*	*SPATA7*
PDE6G	*PGK1*	*RB1*	*RP2*	*SRD5A3*

Gene	Gene	Gene	Gene	Gene
PDE6H	*PHYH*	*RBP3*	*RP9*	*TCTN1*
PDZD7	*PIN1*	*RBP4*	*RPE65*	*TCTN3*
TEAD1	*TIMM8A*	*TIMP3*	*TMEM126A*	*TMEM138*
TMEM216	*UNC119*	*MIR204*	*NBAS*	*PROX1*
TMEM231	*USH1C*	*PLK4*	*PRDM13*	*BMP2*
TMEM237	*USH1G*	*PNPLA6*	*RTN4IP1*	*UCHL3*
TMEM67	*USH2A*	*POC1B*	*SLC25A46*	*LAMA1*
TOPORS	*VCAN*	*PRPS1*	*SPP2*	*MAPKAPK3*
TPP1	*VLDLR*	*TUBGCP4*	*TRNT1*	*PITX1*
TREX1	*VPS13B*	*GPR143*	*FBN1*	*SF3B4*
TRIM32	*WDPCP*	*PLA2G6*	*CEP78*	*GNB3*
TRPM1	*WDR19*	*TRAF3IP1*	*CWC27*	*HMX1*
TSPAN12	*WFS1*	*C10orf11*	*ZGPAT*	*TULP1*
TTC21B	*ZNF408*	*TYR*	*IDH3A*	*BMP4*
TTC8	*ZNF423*	*APOB*	*IFT81*	*EXOSC2*
TTLL5	*ZNF513*	*AGBL5*	*FRMD7*	*HGSNAT*
TTPA	*CEP250*	*ADIPOR1*	*MC4R*	*TUBGCP6*
TUB	*DRAM2*	*CTNNA1*	*MSX2*	

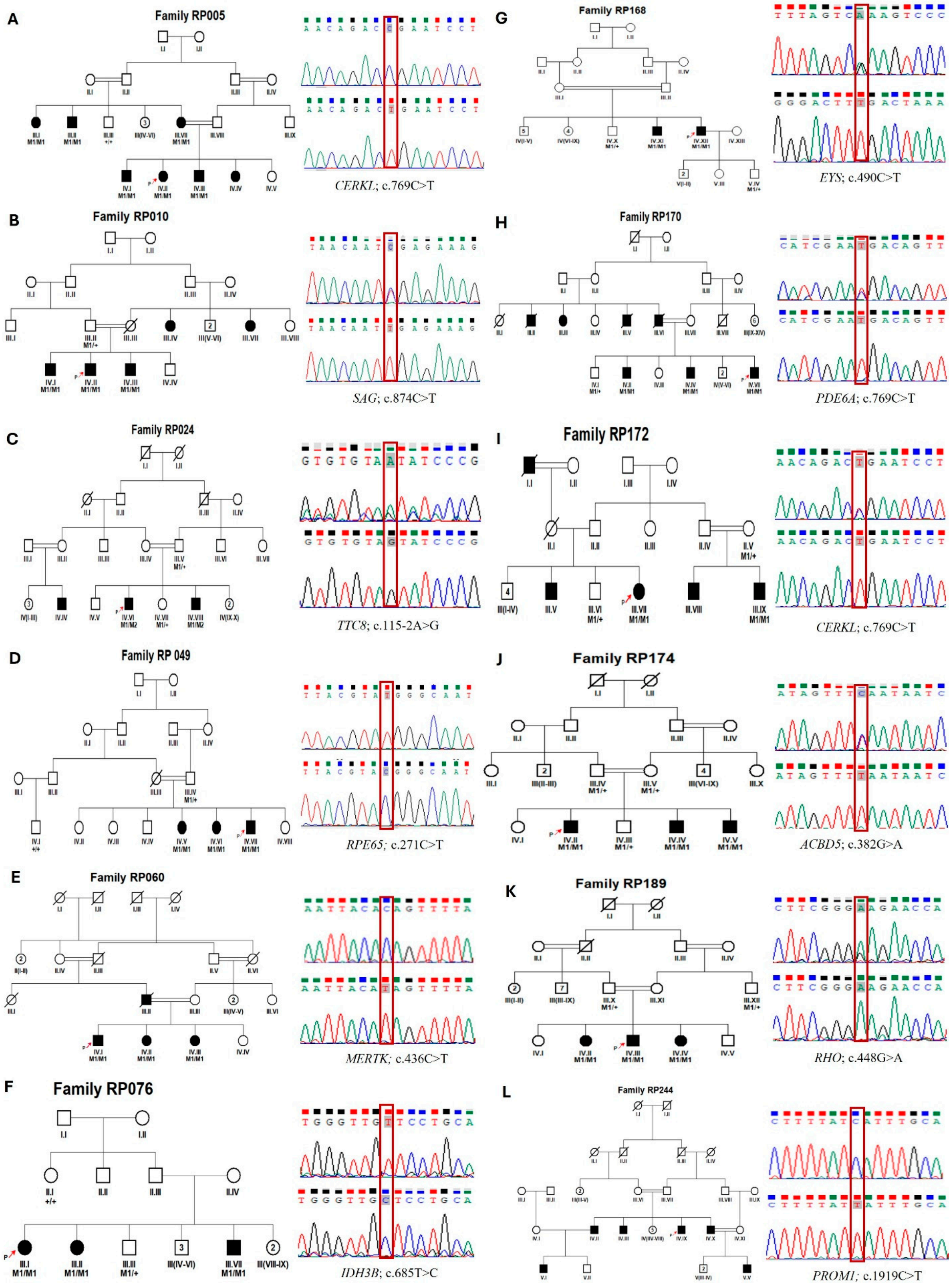
A
Family RP005
CERKL; c.769C>T
B
Family RP010
SAG; c.874C>T
C
Family RP024
TTC8; c.115-2A>G
D
Family RP 049
RPE65; c.271C>T
E
Family RP060
MERTK; c.436C>T
F
Family RP076
IDH3B; c.685T>C
G
Family RP168
EYS; c.490C>T
H
Family RP170
PDE6A; c.769C>T
I
Family RP172
CERKL; c.769C>T
J
Family RP174
ACBD5; c.382G>A
K
Family RP189
RHO; c.448G>A
L
Family RP244
PROM1; c.1919C>T

Supplementary Figure S1. Pedigree analysis, Sanger sequencing validation, and segregation of identified variants in 12 Pakistani families with retinitis pigmentosa. Pedigrees (A–L) represent families RP005, RP010, RP024, RP049, RP060, RP076, RP099, RP168, RP170, RP172, RP174, RP189, and RP244. Squares denote males and circles denote females; filled symbols indicate affected individuals, while open symbols represent unaffected individuals. Double lines indicate consanguineous unions. Arrows mark the probands. Genotypes are indicated as homozygous mutant (M1/M1), heterozygous (M1/+), or wild type (+/+). Representative electropherograms show Sanger sequencing validation of identified variants in affected and unaffected family members, with variant positions highlighted by red boxes. The corresponding gene and nucleotide changes are indicated for each family, including *CERKL* (c.769C>T), *SAG* (c.874C>T), *TTC8* (c.115-2A>G), *RPE65* (c.271C>T), *MERTK* (c.436C>T), *IDH3B* (c.685T>C), *EYS* (c.490C>T), *PDE6A* (c.769C>T), *ACBD5* (c.382G>A), *RHO* (c.448G>A), and *PROM1* (c.1919C>T). The segregation patterns confirm co-segregation of the identified variants with the disease phenotype within each family.
